# Hyperactive delta isoform of PI3 kinase enables long-distance regeneration of adult rat corticospinal tract

**DOI:** 10.1016/j.ymthe.2024.12.040

**Published:** 2025-01-01

**Authors:** Kristyna Karova, Zuzana Polcanova, Lydia Knight, Stepanka Suchankova, Bart Nieuwenhuis, Radovan Holota, Vit Herynek, Lucia Machova Urdzikova, Rostislav Turecek, Jessica C. Kwok, Joelle van den Herik, Joost Verhaagen, Richard Eva, James W. Fawcett, Pavla Jendelova

**Affiliations:** 1 Institute of Experimental Medicine CAS, Department of Neuroregeneration, Videnska 1083, 142 20 Prague, Czech Republic; 2 Institute of Experimental Medicine CAS, Department of Auditory Neuroscience, Videnska 1083, 142 20 Prague, Czech Republic; 3 John van Geest Centre for Brain Repair, Department of Clinical Neurosciences, Cambridge Institute for Medical Research, University of Cambridge, Cambridge CB2 0XY, UK; 4 Institute of Biology and Ecology, Faculty of Science, P.J. Safarik University in Kosice, Srobarova 2, Kosice 041 54, Slovak Republic; 5 Center for Advanced Preclinical Imaging (CAPI), First Faculty of Medicine, Charles University, Salmovska 3, 120 00 Prague, Czech Republic; 6 School of Biomedical Sciences, University of Leeds, Leeds LS2 9JT, UK; 7 Netherlands Institute for Neuroscience, Meibergdreef 47, 1105 BA Amsterdam, the Netherlands; 8 Kings College London, Wolfson Sensory Pain and Regeneration Centre (SPaRC), Guy’s Campus, London Bridge, London SE1 1UL, UK; 9 Institute of Experimental Medicine CAS, Centre for Reconstructive Neuroscience, Videnska 1083, 14220 Prague, Czech Republic

**Keywords:** spinal cord, axon regeneration, signaling, PI3K, pS6, skilled paw reaching, electrophysiology, c-Fos, CST, spinal cord injury

## Abstract

Neurons in the CNS lose regenerative potential with maturity, leading to minimal corticospinal tract (CST) axon regrowth after spinal cord injury (SCI). In young rodents, knockdown of PTEN, which antagonizes PI3K signaling by hydrolyzing PIP3, promotes axon regeneration following SCI. However, this effect diminishes in adults, potentially due to lower PI3K activation leading to reduced PIP3. This study explores whether increased PIP3 generation can promote long-distance regeneration in adults. We used a hyperactive PI3K, PI3Kδ (PIK3CD), to boost PIP3 levels in mature cortical neurons and assessed CST regeneration after SCI. Adult rats received AAV1-PIK3CD and AAV1-eGFP, or AAV1-eGFP alone, in the sensorimotor cortex concurrent with a C4 dorsal SCI. Transduced neurons showed increased pS6 levels, indicating elevated PI3K/Akt/mTOR signaling. CST regeneration, confirmed with retrograde tracing, was evaluated up to 16 weeks post injury. At 12 weeks, ∼100 axons were present at lesion sites, doubling to 200 by 16 weeks, with regeneration indices of 0.1 and 0.2, respectively. Behavioral tests showed significant improvements in paw reaching, grip strength, and ladder-rung walking in PIK3CD-treated rats, corroborated by electrophysiological recordings of cord dorsum potentials and distal flexor muscle electromyography. Thus, PI3Kδ upregulation in adult cortical neurons enhances axonal regeneration and functional recovery post SCI.

## Graphical abstract

**Figure:**
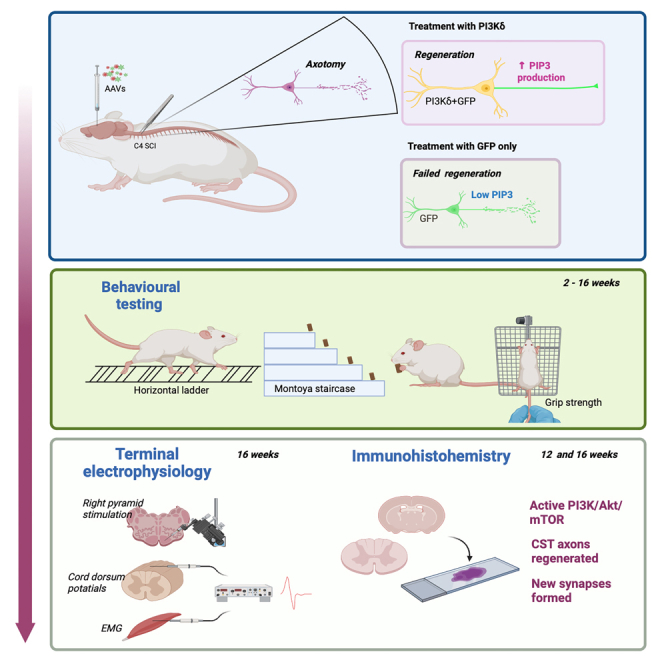

## Teaser

The study identifies a treatment that enables extensive regeneration of motor axons after spinal cord injury. AAV therapy containing PI3Kδ resulted in robust axon growth around the lesion site to areas as far as 1 cm caudally. Adult rats treated with PI3Kδ showed motor-function improvements confirmed by electrophysiological recordings.

## Introduction

Long-distance axon regeneration in the injured adult mammalian spinal cord is an unsolved problem. The intrinsic ability of axons in the brain and spinal cord to regenerate diminishes as neurons mature. There are several mechanisms behind this loss of regenerative ability, notably: (1) reduction in signaling, particularly in the phosphatidylinositol 3-kinase (PI3K)/Akt pathway, known to be crucial in promoting growth; (2) development of neuronal polarity, leading to polarized transport that excludes many growth-related molecules from axons, limiting their regenerative potential; and (3) genetic/epigenetic changes in maturing neurons that affect expression of axon-growth-related molecules.^1^

**Figure 1:**
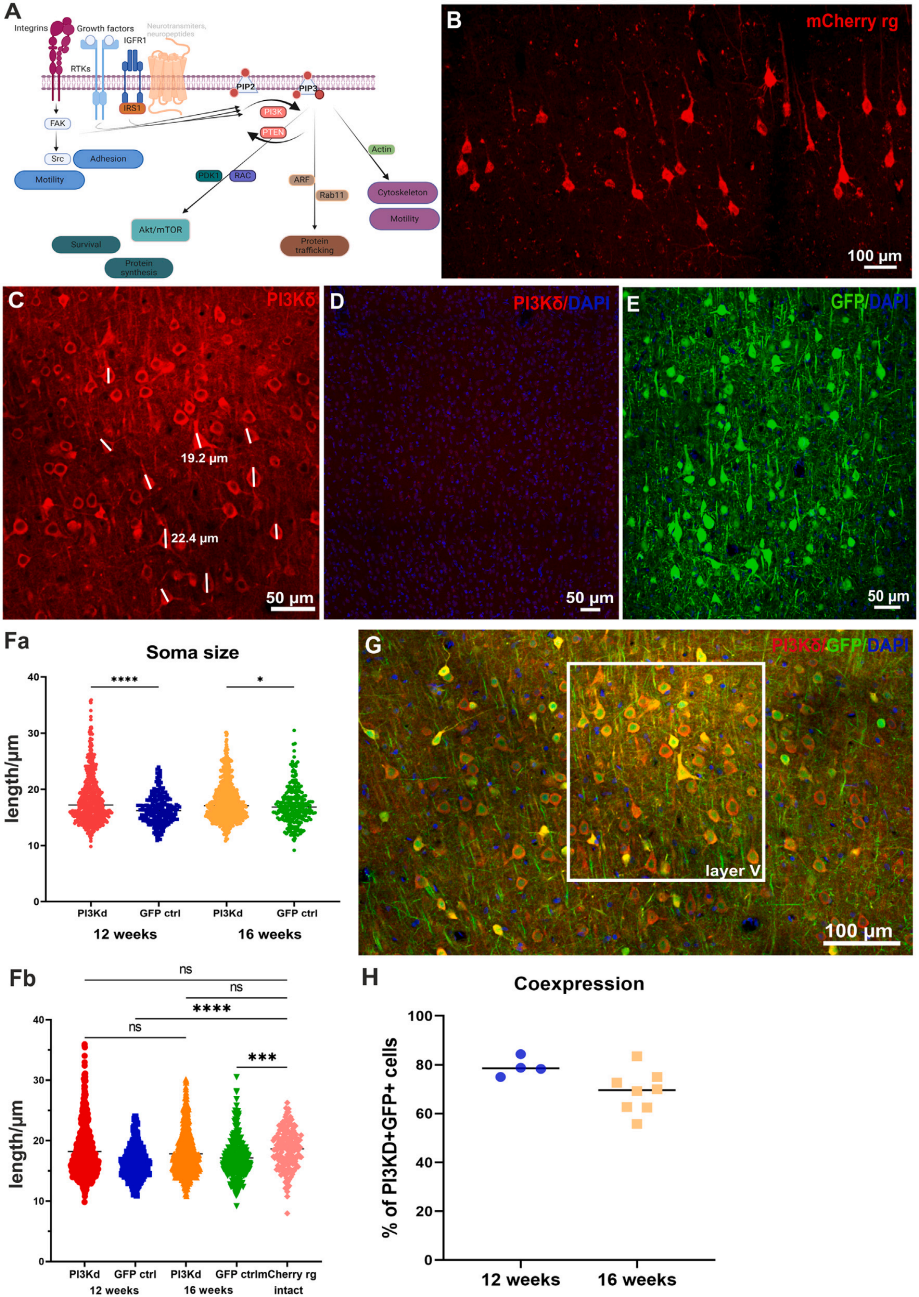
PI3Kδ readily co-expresses with GFP and does not lead to aberrant neural soma morphology PI3Kδ may be activated though several tyrosine kinase receptors (RTKs), after which it generates PIP3 via phosphorylation of PIP2 in antagonizing fashion to PTEN. PIP3 signaling lipid regulates pathways stimulating several domains of cellular biology including survival, protein synthesis, trafficking and motility. Illustration was made using BioRender (A). Lengths of layer V cortical neurons were measured in mCherry^+^ retrogradely labeled intact projecting neurons (B). AAV1-SYN-PIK3CD-treated neurons expressed high levels of PI3Kδ only after treatment (C), since endogenous cortical levels were low (D). Soma lengths were also measured in GFP controls (E). At both 12 and 16 weeks after SCI (F), soma lengths of neurons in control groups were smaller than those overexpressing both mCherry or PI3Kδ (Fa), while no statistical difference was found between PI3Kδ neurons and mCherry^+^ neurons (Fb) Data shown as individual values. Transduced brains were stained against PI3Kδ and GFP at 12 weeks and 16 weeks after surgery to determine co-expression of PI3Kδ^+^ layer V neurons positive for GFP tracer (G). On average, co-expression levels were close to 80% at 12 weeks and declined slightly to around 70% at 16 weeks after cortical injections. Data shown as means from 3-5 brain sections per rat (H). Magnification 40×. One-way ANOVA; ∗∗∗∗*p* < 0.0001, ∗*p* < 0.05.

A key signaling pathway that regulates cell growth and motility^2^ is initiated by PI3K activity, which generates the signaling lipid phosphatidylinositol (3,4,5)-triphosphate (PIP3) (Figure 1A). The levels of PIP3 are tightly controlled and localized by regulating the activity of PI3K, as well as the phosphatases PTEN and SHIP, which dephosphorylate PIP3.^3^ The involvement of PIP3 in axon regeneration has been shown through knockout of PTEN, enabling spinal cord axon regeneration in young animals.^4^^,^^5^^,^^6^ However, in adult animals PTEN depletion has much less effect on regeneration.^6^ The aim of this study was to develop a PIP3-related intervention that is effective in adult mammals. PIP3, produced by PI3K, influences cell motility, protein translation, transport, and various other functions through signaling pathways involving Akt/mTOR, GSK3, and CRMP2, as well as Arf6 GTPase-activating proteins (GAPs) and guanine nucleotide exchange factors (GEFs), and via other proteins that possess PH or FYVE domains.^7^ It has been shown that axon growth is associated with an increase of axonal PIP3 during both development and regeneration,^8^ and manipulation of PI3K to mediate PIP3 increase prevented growth-cone collapse in dorsal root ganglion (DRG) sensory neurons while PTEN facilitated the growth-cone collapse response.^9^ Other studies have revealed that stimulating the PI3K/Akt/mTOR axis results in axon regeneration after spinal cord injury. In one study, the combination of insulin-like growth factor 1 (IGF-1) and osteopontin achieved robust short-distance regeneration by activating the PI3K/Akt/mTOR pathway.^5^^,^^10^

Why does PTEN knockout become less effective in adults? PIP3 levels are high in developing axons but decline sharply as they mature and become electrically active.^8^^,^^11^ In young neurons, PIP3 levels can be increased by reducing its degradation through PTEN deletion. However, this approach becomes less effective in mature neurons, likely due to a decline in PIP3 production by PI3Kα. Although PI3Kα levels remain high in mature neurons, its activation might be reduced, because many PI3K-activating receptors are excluded from mature axons.^11^^,^^12^^,^^13^ This exclusion leads to reduced PIP3 production, making PTEN knockdown less effective in mature neurons.^8^^,^^14^

To elevate PIP3 production in mature neurons and axons, we expressed PI3Kδ as an innately hyperactive PI3K isoform. PI3Kδ is predominantly expressed in cells of the immune system and sensory neurons^15^ and is highly expressed in the developing CNS but downregulated in adults.^15^ Of the known isoforms, expression of PI3Kδ in cortical neurons enabled the most vigorous regeneration in an *in vitro* axotomy assay, and its expression in retinal ganglion cells enabled regeneration of their axons in the adult mouse optic nerve.^8^ Transport of integrins in recycling endosomes was also restored in mature axons, possibly involving the effects of PIP3 on Arf6 GEFs and GAPs controlling endosomal transport.^12^^,^^16^^,^^17^ In contrast, several inhibitors of PI3K isoforms applied to DRGs caused growth-cone collapse^15^ and marked reduction in axon growth when PI3Kα and/or PI3Kδ were blocked.^8^

For restoration of function after spinal cord injury (SCI), repair of the corticospinal tract (CST) is crucial, and this pathway has very low spontaneous regenerative ability. In the present study we used a gene-therapy approach to ask whether expression of PI3Kδ in neurons of the adult rat sensorimotor cortex can enable regeneration of axons of the injured CST. We show that many axons regenerate for distances of over 1 cm, with restoration of sensorimotor behavior and physiological connections to forelimb muscles.

## Results

Pilot experiments indicated that expression of PI3Kδ under the CAG promoter in cortical neurons would enable substantial regeneration of corticospinal axons by 12 weeks after injury. A full experiment was therefore performed, using dorsal column lesions extending through the CST down to the spinal canal with viral vectors injected into the sensorimotor cortex area projecting to the cervical spinal cord where SCI was induced. For the full follow-up experiments with behavior or electrophysiology recordings, we used vectors expressing PI3Kδ under the neuron-specific SYN promoter, which had an expression level comparable with that of AAV1-CAG-PIK3CD (Figures S1A–S1G). This vector was used in combination with SYN-GFP as the axon tracer. We continued our experiments with the SYN promoter because CAG may also induce expression of transgenes in other cell types and co-expression with GFP is low (Figures S1D–S1G). We evaluated the effects of PI3Kδ on axon sprouting in experiments with both vectors. Axon regeneration was sampled at 6, 9, 12, and 16 weeks, with behavioral assessment, retrograde tracing of regenerates, and electrophysiological endpoints at 16 weeks.

### PI3Kδ co-expressed with GFP at high levels and prevented neural soma size reduction

We assessed PI3Kδ and GFP protein co-expression in projection-layer cortical neurons in rats that received a C4 dorsal column crush SCI. Co-transduction with GFP was used to identify transduced neurons and their axons because a construct that includes PI3Kδ, IRES, and a fluorescent protein sequence would vastly exceed the adeno-associated virus (AAV) packaging capacity, which is 4.7 kb. Moreover, tagging PI3Kδ with GFP or any other tag inactivates it. AAV1 injections led to expression of PI3Kδ and GFP in neurons with a soma length of approximately 20 μm (Figures 1C, 1Fa, and 1Fb). The number of PI3Kδ^+^ neurons was determined, from which a percentage of co-expressing neurons emerged by establishing how many of them were also GFP^+^. The injected AAV1 mixture caused an average of 79.7% co-transduction at 12 weeks post surgery and 68.92% at 16 weeks post surgery, which indicated that this method was suitable for tracing axons from PI3Kδ-expressing neurons (Figures 1G and 1H). It has previously been reported that stimulating the pAkt/mTOR pathway using Akt3 overexpression with high-titer viral vectors can lead to disproportionate soma size increases that were associated with several negative effects.^18^ We therefore measured soma lengths of neurons in injured PI3Kδ-treated rats, GFP controls, and retrogradely labeled mCherry^+^ cortical projection neurons in intact rats (Figures 1B, 1C, and 1E). SCI led to reduction in soma size in GFP expressing control neurons, which was partially mitigated by the PI3Kδ treatment (Figures 1Fa and 1Fb). Neurons expressing PI3Kδ fell into a normal soma size range of adult rats (Figures 1C and 1E).^19^

### PI3Kδ increased pS6 expression in transduced cortex

**Figure 2:**
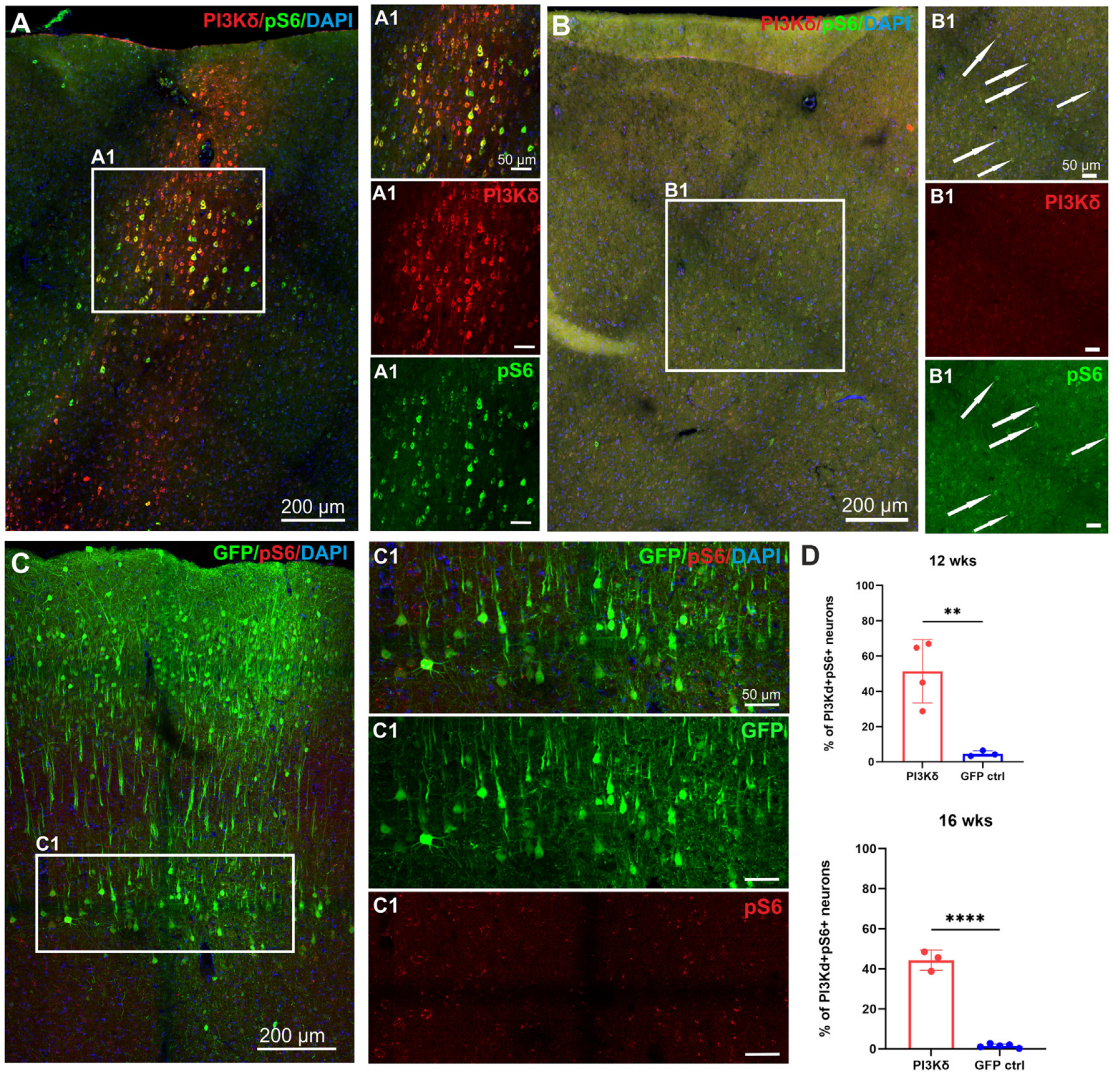
PI3Kδ increases levels of pS6 in transduced cortices Phosphorylation of S6 remains upregulated in PI3Kδ-overexpressing neurons 16 weeks after treatment (A, A1), suggesting active PI3K/pAkt/mTOR pathway. Very low pS6 levels were detected in noninjected cortices (B, B1) and cortices injected with control AAV1 (C, C1). (D) Quantification of pS6 co-expression with PI3Kδ or GFP shows significant increase of S6 phosphorylation at both 12 and 16 weeks after cortical injections. Magnification 20×. Data shown as mean and SEM, Student’s t test; *∗p* = 0.0266, *∗∗∗∗p* < 0.0001.

Phosphorylated S6 protein is a downstream effector of PI3K/PIP3/pAkt/mTOR and is the generally used marker for activity in this signaling pathway. As expected, cortical neuronal pS6 levels were low in our adult rats (Figures 2B–2D), as shown by others.^5^^,^^10^ After transduction with AAV1-SYN-PIK3CD, we observed a large number of strongly stained PI3Kδ^+^pS6^+^ cells within the injection sites of the right hemisphere (Figure 2A), in contrast to the noninjected hemisphere (Figure 2B) and controls (Figure 2C) where there are pS6^+^ cells, but the levels were much lower (Figures 2B1 and 2C1). Approximately half of the analyzed layer V cells expressing PI3Kδ expressed increased levels of pS6. This increase persisted through both 12 and 16 weeks with higher levels at 12 weeks and a slight decrease at 16 weeks (Figure 2D). In conclusion, overexpression of AAV1-SYN-PIK3CD enhanced neuronal pS6 signaling in rats.

### PI3Kδ treatment increased sprouting of axons across midline above the C4 lesion and reduced the number and distance of retraction bulbs from lesion border

**Figure 3:**
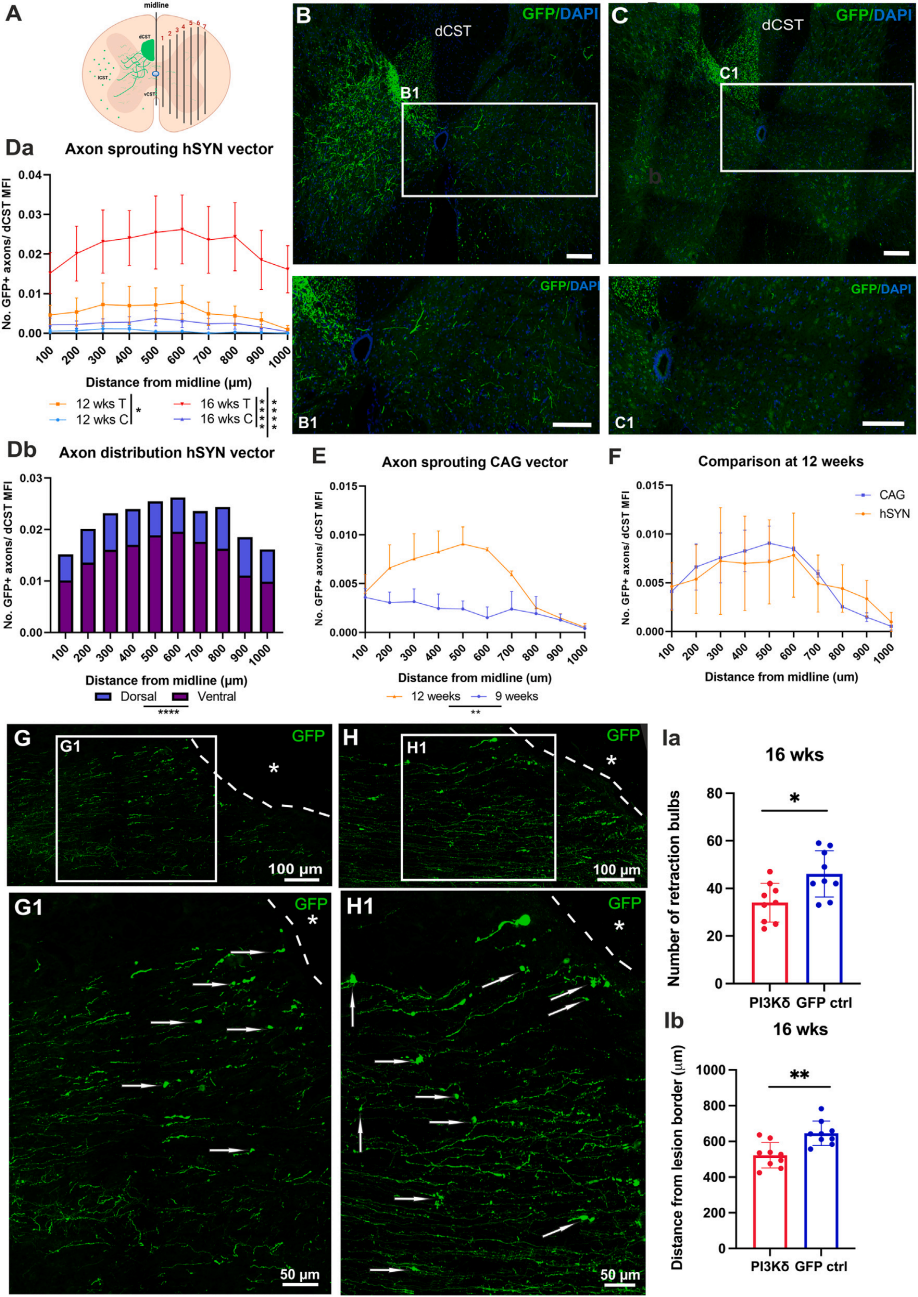
Treatment with PI3Kδ stimulates robust sprouting cranially to lesion site Axon sprouting was determined after treatment with AAV1-SYN-PIK3CD by counting the number of axons in cervical spinal cord above lesion crossing the midline at 100 μm intervals, produced using BioRender (A and Da). Distribution of axons in dorsal and ventral horns was calculated (Db). Representative images of spinal cords from treated animals 16 weeks (B) after SCI and control at 16 weeks after SCI (C). Axon sprouting power was also assessed after treatement with AAV1-CAG-PIK3CD and more axons were seen growing across the midline at 12 weeks when compared to 9 weeks (E). Comparisons between the two different AAV1-PI3KCD vectors used in this study at 12 weeks showed no difference in sprouting power (F). Analysis of axon degeneration was done by counting the number of retraction bulbs (white arrows) and their distance from the lesion border (dashed line and asterisk) in AAV1-SYN-PIK3CD treated rats (G, G1) and GFP controls (H, H1) at 16 weeks after SCI and AAV1 injections. Treatment with PI3Kδ reduced the number of retraction bulbs observed in dCST (Ia) and reduced the length of axon degeneration (Ib). Individual values were plotted; one-way ANOVA and t test with Mann-Whitney post hoc test were used. For axon sprouting analysis data was shown as mean and SEM. *∗p* = 0.019, *∗∗∗p* < 0.001, *∗∗∗∗p* < 0.0001. dCST, dorsal corticospinal tract. Scale bars 200 μm unless otherwise indicated.

We asked whether AAV-hSYN-PIK3CD treatment induces sprouting of axons in areas above the lesion, where they are intact. We analyzed spinal cord cross-sections and counted axons that sprouted across the midline contralaterally to the labeled dorsal CST (dCST) (Figure 3A) after treatment (Figures 3B and 3B1) and compared them to GFP-only controls at 12 and 16 weeks (Figures 3C and 3C1). There was extensive sprouting of axons across the midline in the treatment groups, with many more at 16 weeks (Figure 3Da). In contrast, only a small number of fibers crossing the midline were found in the control groups (Figures 3C, 3C1, and 3Da). We also analyzed the distribution of axons and found that the majority of axons entered the intermediate and ventral horn (VH) area (Figure 3Db). We conducted part of the study with AAV vectors containing the CAG promoter to validate and compare time-dependent effects of PI3Kδ packaged into different vectors on axon growth. We therefore analyzed sprouting power in groups treated with AAV1-CAG-PIK3CD and saw a significant increase in the number of axons that sprouted across the midline at 12 weeks when compared to 9 weeks after treatment (Figure 3E) and no difference in treatment power at 12 weeks between AAV1-CAG-PIK3CD and AAV1-hSYN-PIK3CD (Figure 3F).

Further, we wanted to determine whether PI3Kδ treatment had a similar effect on axons as it had on neural soma. To this end, we counted the number of retraction bulbs (RBs) and their distance from the spinal cord lesion border in treated rats (Figures 3G and 3G1) and controls (Figures 3H and 3H1) 16 weeks after treatment and SCI. Indeed, we found fewer RBs in treated animals, and these were at a shorter distance from lesion border in treated rats when compared to controls (Figures 3Ia and 3Ib).

### Overexpressing PI3Kδ leads to the growth and sprouting of many axons over long distances below the C4 lesion

The main endpoint of the study was regeneration of corticospinal axons through and below spinal injuries. To evaluate the number of axons that grew below the lesion site, we counted them in every other 20-μm sagittal section. Lesions produced a cavity in the dorsal cord reaching down to or beyond the central canal (CC), roofed by a fibroblastic/meningeal structure. In this study, we ensured that only animals with complete lesions were analyzed, which we determined by MRI of whole spinal cord segments and protein kinase Cγ (PKCγ) staining in cross-sections from lumbar spinal cords (Figures S1H–S1K). Moreover, using MRI, we analyzed and compared lesion sizes in treated and control rats and did not find a significant difference (Figure S1L).

**Figure 4:**
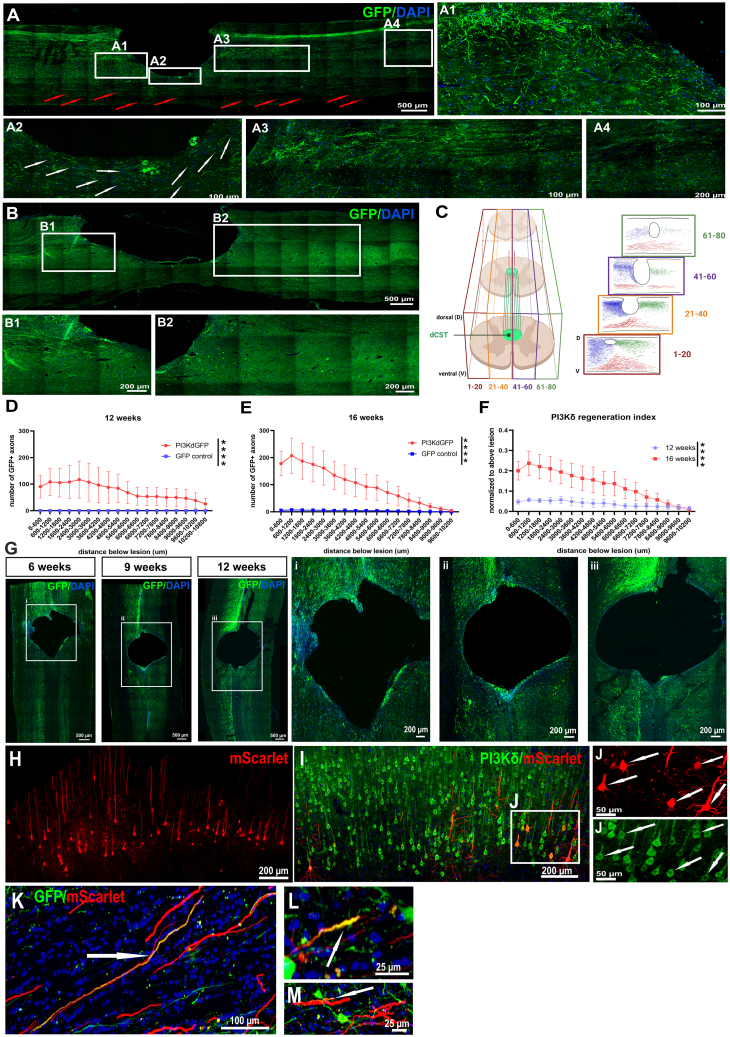
Axon count analysis,growth pattern reconstruction, and retrograde tracing Axon count below lesion was evaluated in every other 20-μm spinal cord sagittal section in treated (A) and control (B) animals. Sums of axons 12 and 16 weeks after SCI were calculated for each 600 μm and their distribution up to 1 cm depicted (D and E). Regeneration indices were determined according to axon count normalized to values above lesion and compared between 12 and 16 weeks after SCI and treatment (F). White arrows annotate regenerating axons growing around the lesion borders, while red arrows indicate intact ventral CST axons not included in analysis. A reconstruction of axon growth in the lesion area and below was drawn using BioRender by approximate drawing of GFP^+^ axons and neurites. Blue color was assigned to those found cranially to lesion and extending, red color was assigned to axons and neurites in the ventral part of sections, and green color represents neurites residing in the dorsal half of the cord caudal to the lesion border (C). Drawing was inspired by Han et al.^20^ Axon analysis after PI3Kδ treatment under CAG promoter was assessed in spinal cords with frontal plane of sectioning from rats treated with AAV1-CAG-PIK3CD + AAV1-CAG-eGFP vector mixture at 6, 9, and 12 weeks post injury and cortical injections (G). Axons reach the lesion border and start bypassing the lesion laterally to the cavity and beyond (Gi, Gii, Giii). Retrograde tracing with AAV9rg-hSYN-mScarlet revealed several back-labeled regenerating cortical neurons (I and J), and their numbers were much lower than traced corticospinal motor neurons in intact animals (H). An example of a section with double-labeled mScarlet^+^GFP^+^ regenerated axons (K, L, and M) annotated with white arrows. Data shown as mean and SEM, paired t test; ∗∗∗∗*p* < 0.0001.

At the rostral end of lesions there were many transected axons, which were in contact with the lesion edge (Figure 4A1). In treated animals, many cut axons could be seen to have sprouted processes around the lesion margins ventrally (Figure 4A2) and laterally (Figure 4G). The peri-lesional regeneration was progressive, with a few axons at the rostral end of the lesions at 6 weeks (Figure 4Gi), with increasing numbers having regenerated progressively further around the lesions and down the cord at 9, 12, and 16 weeks (Figures 4Gii, 4Giii, and 4A). In GFP controls few axons sprouted (Figures 4B, 4B1, and 4B2). A reconstructed lesion from an AAV1-SYN-PIK3CD-treated rat, sectioned sagittally, is shown in Figure 4C. Regenerating axons were seen meeting the lesion and then growing around the lesion margin. At the caudal margin of the lesion, an accumulation of regenerated axons was seen as the peri-lesional axons collected (Figures 4A3 and 4C). From this bundle, many axons were seen to grow on in the dorsal cord (Figures 4A3 and 4A4). These had the typical thin meandering morphology of regenerated axons. A progressively decreasing number of these axons was seen to 1 cm below the lesions. The number of axons at the caudal lesion margin was on average 100 at 12 weeks and 200 at 16 weeks (Figures 4D and 4E). Comparing labeled axon numbers rostral and caudal to the lesions the regeneration indices were 0.1 and 0.2, respectively (Figure 4F). A complication in quantifying regeneration in dorsal column lesions is that the ventral CST (vCST) is not cut, and the axons of this pathway may sprout after injury. It is easy to distinguish the unlesioned axons because they are straight with a large diameter compared to regenerates (annotated with red arrows in Figure 4A). However, the sprouts are difficult to distinguish from those of regenerated axons. This is illustrated in Figure 4C, where sprouted ventral axons are shown in red. The position of regenerated CST axons in the dorsal cord and their continuity with the knot of axons at the caudal end of the lesion makes it easy to identify regenerates for 0.5 cm caudal to the lesion (Figure 4A3). For a further 0.5 cm, a steadily decreasing number of labeled axons with the morphology of regenerates in the same position was observed (Figure 4A4). We identified these as regenerated CST axons, although with lower confidence than for the more rostral 0.5 cm. In cross-sections at approximately 1 cm below the lesion where there are a few regenerated axons, we observed axons in intermediate and ventral gray matter near the CC (Figures S2A and S2B). Additionally, the growth pattern in rats treated with AAV1-CAG-PIK3CD that were sectioned frontally was observed, and axons also had the tendency to bypass the lesion laterally at 9 and 12 weeks after injury, with more found at the longer time point (Figures 4Gii and 4Giii).

To verify that some of the growth we detected was indeed regeneration of severed axons beyond the lesion, we injected AAV9rg-hSYN-mScarlet intraspinally below the C4 lesion within the C5 segment 16 weeks after treatment. Two weeks later, brains and spinal cords were dissected and studied. Injection sites showed intense mScarlet staining in cell bodies below the lesions and their axons; there was no cell body staining within or above the lesion. Brain coronal sections were stained for PI3Kδ and mScarlet to visualize co-localization in layer V cortical neurons. In intact control animals, mScarlet spinal injections labeled almost all the neurons in the projection layers in the sensorimotor cortex (Figure 4H). In lesioned and PI3Kδ-treated rat brains (Figures 4I and 4J), we saw small numbers (approximately 50 per animal) of retrogradely labeled cortical neurons whose axons had presumably regenerated. Additionally, staining for GFP and mScarlet was performed whereby a small number of GFP^+^mScarlet^+^ cortical axons were found in the subcortical white matter, confirming regeneration (Figures 4K–4M).

GFP^+^ axons in spinal cross-sections below the lesion site were seen mainly in areas around the CC, in both ventral horns (VHs) with predominance in VH contralateral to the injected brain hemisphere, and in contralateral dorsal horn (DH) (Figures S2A, S2B, and S3), which we believe reflect the lateral growth pattern of regenerated and/or sprouted axons from the dCST and the normal CST pathway, since CST axons in rodents terminate in the DH.^21^^,^^22^^,^^23^^,^^24^ Axons which were seen on the ipsilateral side of spinal cords most likely sprouted above the lesion site and crossed the midline before continuing to grow caudally (Figure 3).

### GFP-labeled axons below the lesion formed excitatory synapses with spinal neurons

**Figure 5:**
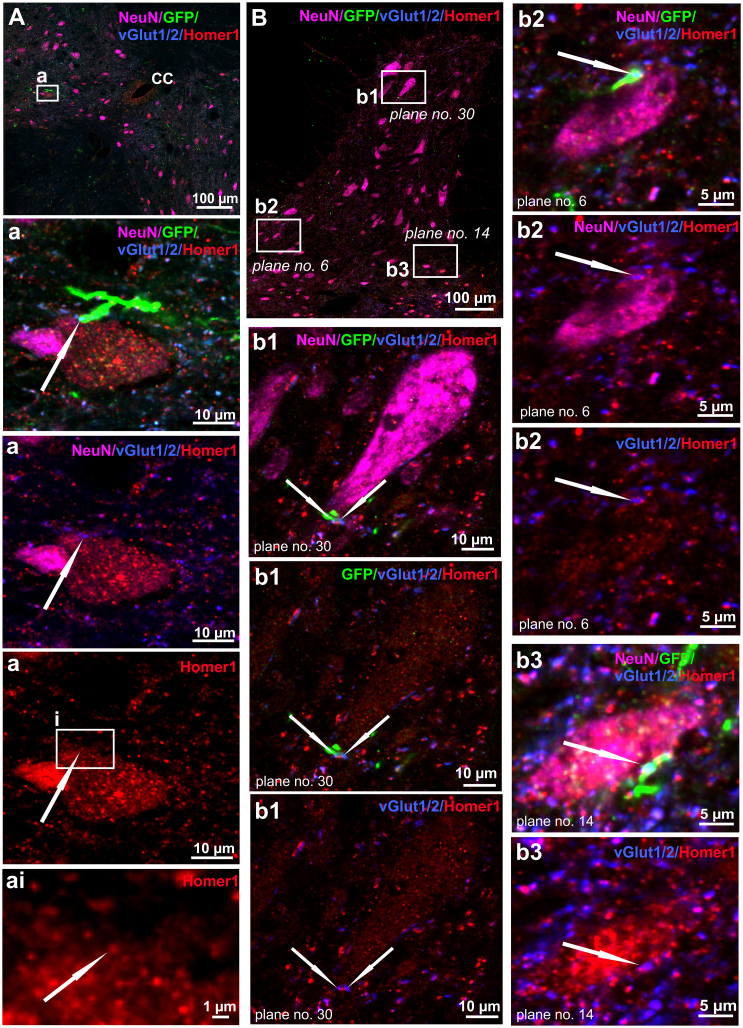
Mature synapses formed between regenerated axons and spinal neurons below spinal cord injury In 20-μm cross-sections from area beyond 1 cm below injury site, regenerated axons marked with GFP were found to form mature excitatory synapses (vGlut 1/2 and Homer1) with neurons (NeuN) at distances of more than 1 cm caudally from spinal lesion. Examples are shown in areas of the intermediate zone near the central canal (A, Aa, Aai, Bb3) and ventral horn (B, Bb1, Bb2). Z-stack images were acquired with confocal spinning-disk microscope at high magnification (40× and 63×), but only single-plane images are shown to highlight the new synaptic connections between regenerated axons and neurons (annotated with white arrows).

Because we found a considerable number of axons caudal to lesion with branches into dorsal, intermediate and ventral gray matter, we examined spinal cord cross-sections and looked for markers of excitatory synapses in the GFP-labeled axons around 1 cm below injury. Indeed we detected puncta of vGlut1/2, a marker of excitatory synapses, co-localizing with GFP^+^ axonal fibers closely associated with spinal neurons (NeuN). Furthermore, these were found to be part of mature synapses additionally identified with a postsynaptic marker, Homer1. Regrown axons formed synapses with many neurons in spinal cord gray matter, including the intermediate zone near the CC (Figures 5A and 5Bb3) and ventral horns (Figures 5B, 5Bb1, and 5Bb2). This shows that newly formed axons establish synapses with targets below the lesion.

### Overexpressing PI3Kδ improved behavioral outcomes and was motor function specific

**Figure 6:**
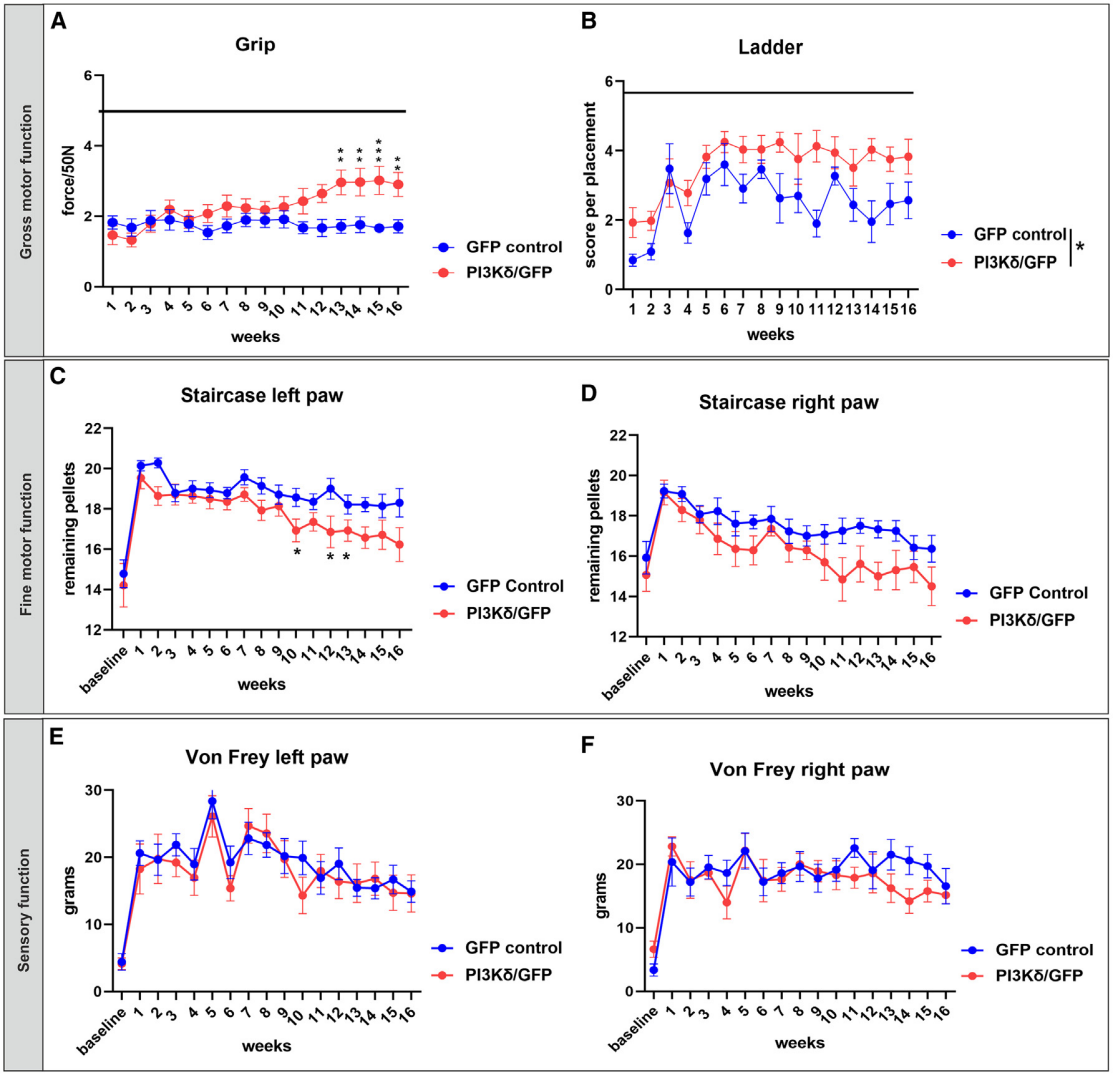
Fine and gross motor functions were improved after PIK3CD treatment Lister Hooded rats were trained for 2 weeks and baseline recorded, followed by lesion induction and cortical injections of vectors. After a 1-week break, rats were tested once a week for 16 weeks to determine changes in gross (A and B) and fine (C and D) motor function. Rats expressing PI3Kδ (*n* = 15) outperformed GFP controls (*n* = 14) in grip strength, pellet reaching, and ladder crossing. In grip assessment, Mixed-model two-way ANOVA revealed overall significance (∗∗*p* = 0.002) with Bonferroni post hoc test significant differences at weeks 13–16 (A). Ladder crossings were scored on a scale of 0–6, which captures errors in front paw placements. Mixed-model two-way ANOVA revealed significant difference in performance between treated and control rats (∗*p* = 0.0295) (B). Mixed-model two-way ANOVA and t test were used to calculate overall significance in skilled left paw reaching (∗*p* = 0.0297) with differences in individual weeks 10, 12, and 13 (C). Assessment of the right paw fine motor movement did not reach significant difference, with *p* = 0.0514 (D). Sensory von Frey test showed no difference between groups (E and F). Data are shown as mean and SEM.

Treated and control rats were behaviorally tested once per week for 16 weeks after injury following a 1-week recovery break. Motor tests included horizontal ladder crossing, grip strength, and skilled paw reaching using the Montoya staircase. Motor recovery started at week 6 and, gradually, treated rats outperformed controls in all motor tests. The best performances were recorded in the last period from 10 weeks onward of testing (Figures 6A–6C). Skilled paw reaching was significantly better in the left paw, although we also recorded improvement in the right paw regarding the number of pellets eaten (Figures 6C and 6D). This probably reflects the fact that regenerated axons were seen on both sides of the cord below the injury. We included a sensory von Frey test to rule out hyperalgesia/allodynia and saw very slight and insignificant restoration of sensation with time, which was still far above baseline, as we expected. This confirmed that our treatment strategy was motor pathway specific, and the minimal sensory function improvement was likely due to spontaneous recovery (Figures 6E and 6F).

### Electrophysiology recordings confirmed functional improvements

**Figure 7:**
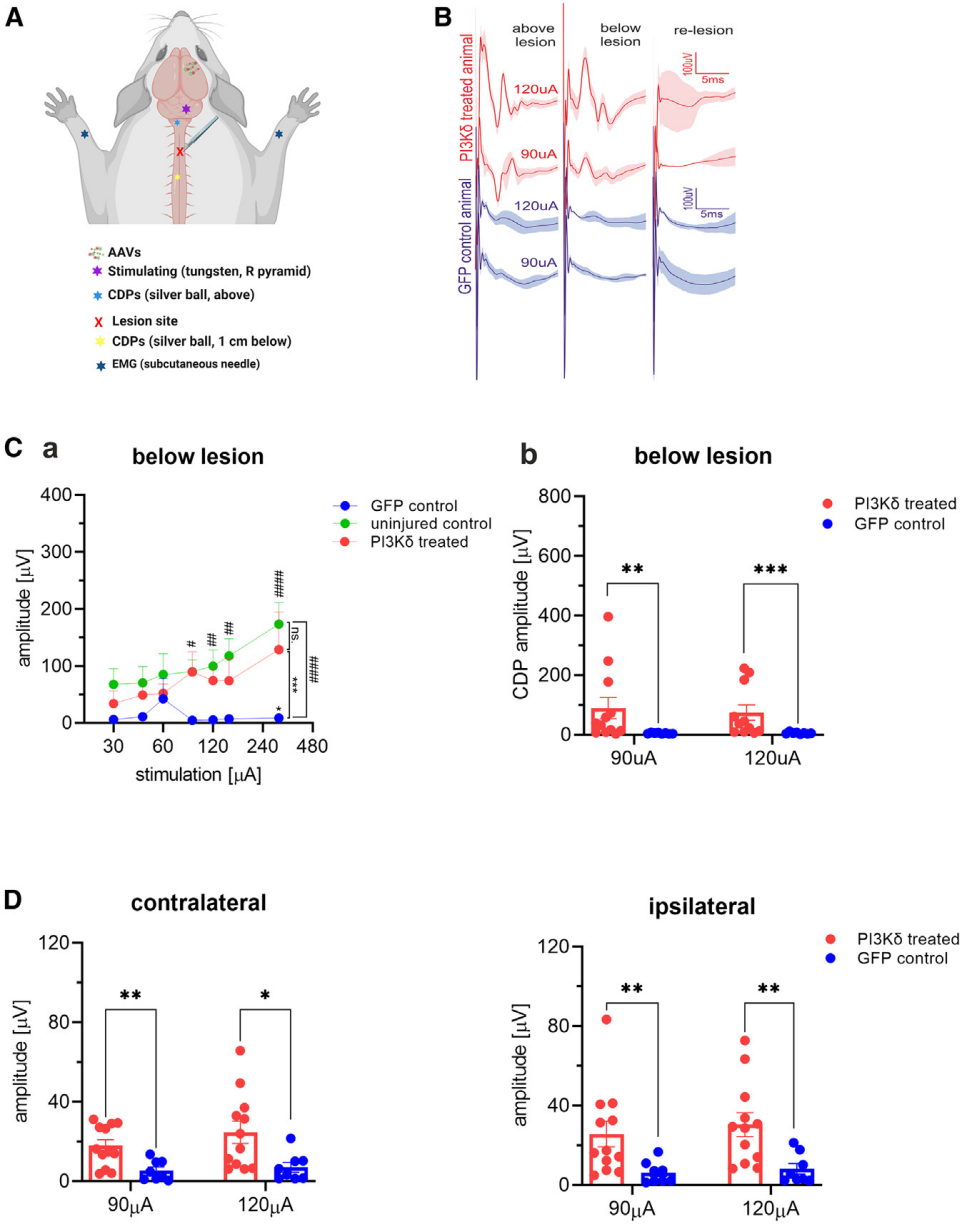
Electrophysiology revealed functional recovery of PI3Kδ-treated rats Improved connectivity in PI3Kδ-treated rats (*n* = 12) was confirmed by electrophysiological recordings, in which the right pyramid was stimulated using a tungsten electrode (five square-wave pulses at 300 Hz) with increasing current amplitudes (30–300 μA). CDPs were measured on spinal surface laterally to midline with silver ball electrodes above and below lesion. EMGs of distal forepaw muscles were recorded concurrently. Schematic produced using BioRender (A). Examples of CDP responses in treated and control rats at 90 μA and 120 μA (B). Recordings were compared with healthy uninjured rats (*n* = 6) and GFP control rats (*n* = 9) (C). Significant differences were found between groups with two-way ANOVA and Sidak’s post hoc tests. No significant difference (ns) between uninjured and PI3Kδ-treated rats was found 1 cm below lesion as opposed to controls, which elicited significantly smaller responses than both treated and uninjured rats (Ca). Significantly stronger responses were recorded in PI3Kδ-treated rats compared to GFP controls at 90 μA and 120 μA stimulating currents (Cb). EMG recordings in distal flexor muscles showed significantly better responses at 90 μA and 120 μA stimulating currents (D). Data shown as mean and SEM. *∗*^,#^*p* < 0.05; *∗∗∗*^*,*###^*p* < 0.001; *∗∗∗∗*^,#####^*p* < 0.0001. CDPs, cord dorsum potentials; EMG, electromyography.

To assess electrophysiological connectivity, spinal cord dorsal surface responses were measured in a different set of animals, as in previous studies,^25^ 1 mm laterally from the midline, 1 cm above the lesion (Figures 7A, 7B, and S4), and 1 cm below the lesion (Figures 7A–7C) in treated, uninjured, and control animals 16 weeks after treatment. The right pyramid was stimulated by an implanted tungsten needle electrode with increasing current amplitudes, and cord dorsal potentials (CDPs) were measured with a silver ball electrode. After recordings, we confirmed that re-lesioning the cord led to loss of signal. Significantly higher potentials were measured in AAV1-SYN-PIK3CD-treated rats when compared with their AAV1-SYN-GFP-treated controls but were not significantly different from uninjured animals (Figures 7Ca and 7Cb). In addition to CDPs, we also measured electromyography (EMG) potentials in distal forepaw muscles of both left and right paws through transcutaneous needle electrodes with stimulation in the right pyramid. We recorded significantly greater responses in both contralateral and ipsilateral paws to the treated hemispheres, corroborating our axon sprouting analysis results (Figures 7D and 3).

### Neuronal activity in spinal neurons was upregulated after stimulation of PI3Kδ-treated rats

**Figure 8:**
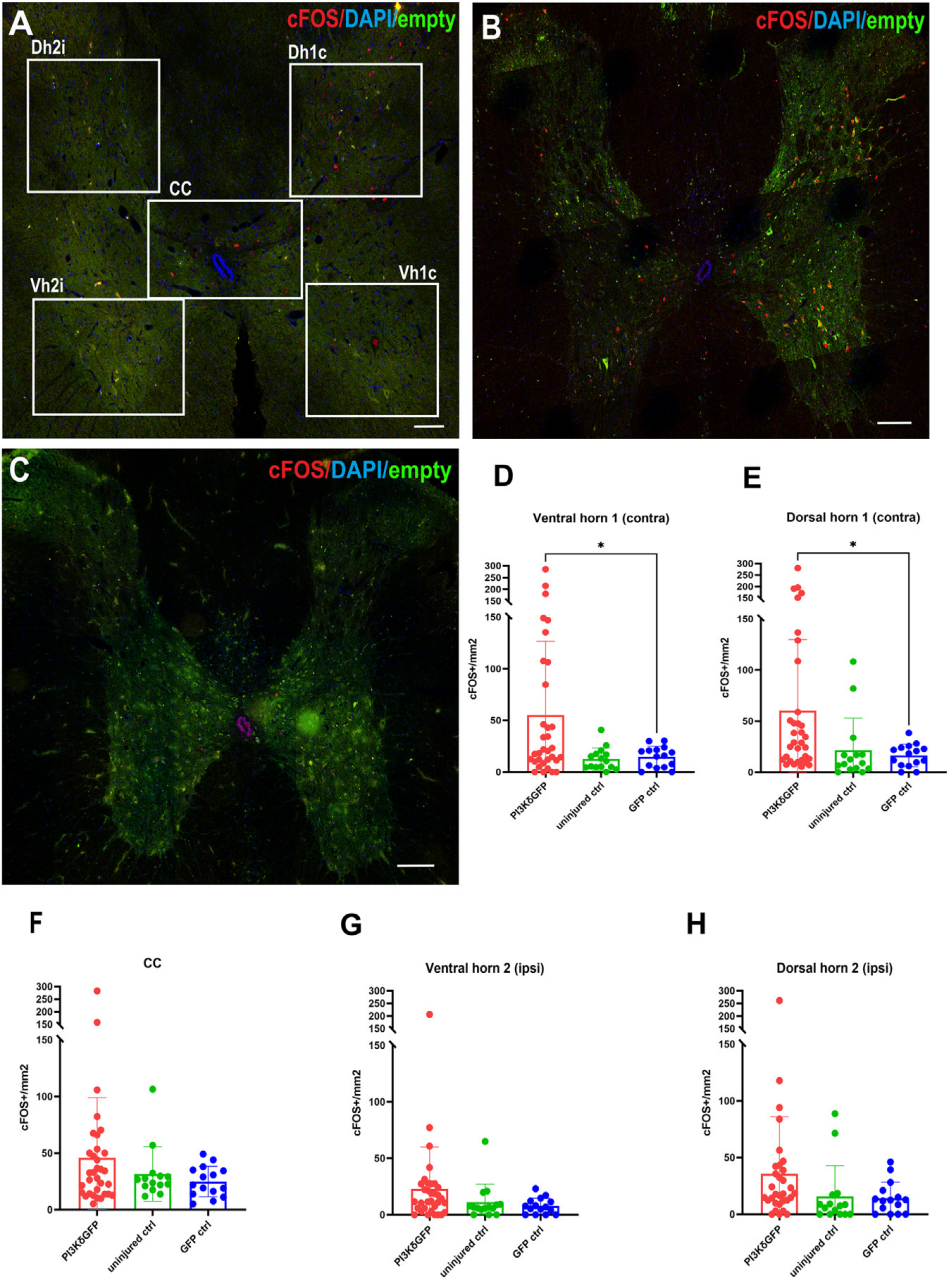
Neuronal activity marker increase in treated and stimulated rats cFOS^+^ nuclei were counted in five regions of 20-μm spinal cross-sections 1 cm below lesion. Representative images show cFOS activation pattern in stimulated uninjured (A), PIK3CD-treated (B), and GFP control (C) rats. Differences were determined with one-way ANOVA in both contralateral ventral horn (Vh1c) and contralateral dorsal horn (Dh1c) where the density of cFOS+ nuclei is higher in treated rats as opposed to GFP controls (*p* = 0.0433 and *p* = 0.0288, respectively) (D and E) No statistical difference was found in the area adjacent to central canal (CC) between treated rats (*n* = 11), control rats (*n* = 5), and uninjured shams (F). No statistically significant difference was found on the ipsilateral side (G and H). Values from individual sections (three per rat) were plotted + SEM. Scale bars, 200 μm.

To discover whether electric stimulation elicited a molecular response indicative of neuronal activity in postsynaptic spinal cord neurons, expression of the early immediate gene marker of neuronal activity, cFOS, was evaluated. After recordings, animals were kept for an additional 2.5 h before perfusion so that cFOS analysis could be carried out in the areas above (Figure S4) and below (Figure 8) the lesion. This timing gave optimum signal in regenerated animals. Numbers of cFOS-positive nuclei were counted around the CC (Figure 8F) and in VH and DH contralaterally (Figures 8D and 8E) and ipsilaterally (Figures 8G and 8H) to the treated hemisphere. Increased expression density of nuclear cFOS was observed both above and below the C4 lesion in PI3Kδ-treated rats. Significant differences were found in VH and DH contralaterally to the treated hemisphere in AAV1-SYN-PIK3CD-treated animals (Figures 8B–8E) when compared to their controls (Figure 8C) but not when compared to uninjured rats (Figures 8A–8E).

## Discussion

The aim of this study was to test the ability of PI3Kδ to enable axon regeneration of adult CST axons in rat spinal cord after injury and to restore sensorimotor function and physiological connectivity. The study followed on from previous studies that indicated that intervention in the PI3K/PIP3/Akt/mTOR pathway through PTEN deletion could induce good corticospinal regeneration in young animals but much less in adults,^6^ while expression of Akt3 could induce axon regeneration but without behavioral recovery and with induction of seizures.^18^ Our strategy was to enhance PIP3 generation through expression of PI3Kδ, a hyperactive form of PI3K, which has enhanced cortical axon regeneration *in vitro*.^8^ AAV1-mediated delivery of PI3Kδ resulted in expression in many output neurons in the sensorimotor cortex, protecting neurons from atrophy but without causing neural soma hypertrophy, which is commonly found in neurons with an overly stimulated Akt/mTOR axis.^26^^,^^27^^,^^28^ Increased S6 phosphorylation in these neurons indicated that the kinase was activating downstream signaling, but PIP3 has widespread effects on proteins carrying the PH or FYVE domains, so the effects of PI3Kδ expression were likely not limited to the canonical effects of the PI3K/Akt/mTOR pathway, which are described as regulating mainly protein synthesis in the soma. CST axons were traced by co-transduction of GFP in animals with C4 dorsal column lesions and were seen regenerating around the margins of the lesions as early as 6 weeks after SCI, with numbers progressively increasing and regenerating further down the cord at 9, 12, and 16 weeks. By 16 weeks many axons were seen that had regenerated past the lesions and on down the cord, with numbers steadily decreasing to 1 cm, where our sagittal tissue sectioning reached. CST regeneration was additionally confirmed by retrograde tracing of corticospinal layer V motor neurons. Even beyond the 1-cm distance, we detected some GFP-labeled axons in spinal cord cross-sections, which associated with spinal neurons and formed mature synapses with them. Treated animals recovered in grip strength, skilled paw reaching, and ladder-walking tasks. Stimulation in the pyramid showed electrical connectivity from the pyramids to below the lesions, seen in cord dorsal electrode and distal forelimb EMG recordings. Electrical stimulation led to an increase in cFOS nuclear signal in the spinal cord gray matter both above and below the lesion site. Overall, the study shows that PI3Kδ expression stimulates extensive CST regeneration, electrophysiological connectivity, and functional recovery.

### PI3K signaling and CST regeneration

PI3K and PIP3 signaling has been associated with axon growth in many studies, while inhibitors of PI3K are blockers of axon growth. Raised PIP3 levels have been implicated in CNS axon regeneration after PTEN deletion or inhibition.^5^^,^^29^ PTEN dephosphorylates PIP3 to PIP2, reversing the action of PI3K. PTEN knockout therefore prevents PIP3 dephosphorylation, thus increasing its level and regeneration of axons in the optic nerve, CST, and other CNS pathways. However, the regeneration-inducing effect of PTEN deletion falls off with age.^6^ *In vitro* studies with PIP3 staining have shown that PIP3 levels in the processes of cortical neurons fall with age, and PTEN knockout is less effective at increasing them. Neurons contain plentiful PI3Kα, another PI3K isoform of the same class,^8^ implying that there is little activation of this enzyme in mature axons and little PIP3 generation. PTEN deletion in the absence of PIP3 generation will therefore have limited effect. For this reason, a hyperactive form of PI3K, PI3Kδ, was transduced into cortical neurons. It behaves similarly to PI3Kα that has a mutation, H1047R, which enhances dynamic events in the natural activation process,^30^ resulting in enzyme activation and PIP3 generation.^8^^,^^31^ Expression of PI3Kδ in mature cortical neurons enhances PIP3 levels in growth cones and enables their axons to regenerate after laser axotomy. It was effective in retinal ganglion cells for stimulation of axon regeneration after optic nerve crush both in transgenic mice and using an AAV2-mediated delivery approach.^8^

PIP3 signals through the Akt pathway, with many downstream targets including protein translation via mTOR. Here, we used increased S6 phosphorylation in PI3Kδ-overexpressing cortical neurons downstream of PI3K/Akt/mTOR as a marker PI3Kδ activity, similar to previously published results.^5^^,^^10^ It is also probable that local translation of mRNAs in axons is increased by PIP3/Akt signaling, with positive effects on axon regeneration. However, PIP3 has widespread effects on proteins with PH and FYVE domains, which play a role in membrane localization and trafficking. Some key cell-surface receptors such as integrins travel into axons in Rab11-positive endosomes found in the cell body and dendrites, but maturational changes lead to polarization of transport within CNS neurons, leading to many molecules including integrins and Rab11 endosomes being excluded from axons.^11^^,^^32^ *In vitro* experiments showed that PI3Kδ expression restores anterograde transport of Rab11 vesicles and integrins into mature axons and promotes axon regeneration of cortical neurons.^8^

Upregulation of the neuronal mTORC1 complex, activated specifically by Akt1 or Akt2, leads to an increase in Paladin levels and upregulation of S6 phosphorylation. This cytoskeleton-modulating protein regulates developmental axon extension and localizes in cortical neuron somata, but to a lower degree than in axon and growth-cone domains. Overexpression of Paladin results in the formation of multiple axons in mTORC1-dependent fashion concurrently with its translation within studied neurites.^33^ Activation of the Akt pathway through expression of Akt3 produced a somewhat different result compared to our study. Strongly overexpressed neuronal Akt3 with high-titer gene therapy led to extensive CST axon regeneration but without motor recovery. This treatment also led to seizures that were associated with increased neural soma size and megalencephaly of the injected hemisphere. This was prevented when the authors adjusted the AAV titer.^18^ In our study, we observed injury-induced neural soma atrophy in GFP controls upon comparison with mCherry retrogradely labeled intact neurons, while neurons treated with PI3Kδ remained within normal size range. We do not believe that applying AAVs concurrently with SCI had a meaningful effect on neural soma size because it takes approximately 2 weeks for transgenes to start expressing, therefore we consider this to be a clinically relevant approach with treatment after SCI. However, preserving neural soma morphology may have had an effect on cortical synaptic activity, which in turn may have promoted axon regrowth, as it is known that electrical stimulation enhances CST tract repair.^34^^,^^35^^,^^36^ In fact, an *in vitro* study using primary cortical neurons showed that electrical stimulation with alternating current led to increased activity of the PI3K/Akt pathway, neurite outgrowth, and activation of regeneration-associated genes, including *Gap43* and *Fos*. All these regeneration indicators were strongly reduced when PI3K inhibitor LY294002 was used.^37^ However, data from a recent study where the authors used chronic activation of CST neurons demonstrated no improvements in front limb motor function nor increased sprouting across midline in mice that received unilateral pyramidotomy.^38^

### Axon regeneration in this study

Regenerating axons were seen mainly in regions of gray matter surrounding the lesions, as is typically the case in both mouse^4^^,^^20^ and rat^18^^,^^39^ CST regeneration studies. Here, we show progressive axon regrowth demonstrated at several time points after SCI induction with concurrent treatment application as well as reduced CST axon retraction in PI3Kδ-treated rats, which revealed neuroprotective effects as seen in analysis of RBs. In general, axons followed the edge of the lesions ventrally and laterally, usually within 200 μm. Having passed around the lesion cavities, they collected at their caudal edge, where many fine axons with the typical fine meandering anatomy of regenerated axons were found randomly oriented. From here, most of the axons grew in a more directed fashion in a caudal direction where a band of axons was seen within approximately 0.25 mm from the dorsal surface, their numbers steadily diminishing with distance from the lesion. Axons grew around the whole extent of lesions, and therefore axons caudal to the lesion were in both sides of the cord. Axons were seen mainly in the contralateral DH, which reflects the lateral pathway of regeneration, but some grew around the injury and into the ipsilateral side of the cord. Our lesions were dorsal, so the vCST was not cut. Thick straight unlesioned axons were therefore seen in the ventral cord when sectioned sagittally. Between this group of clearly unlesioned axons and the clearly regenerated axons there is a region of gray matter containing many fine processes, which could be branches from the regenerated axons or the unlesioned vCST; neurites in this region were not counted when they were seen to emerge from the vCST. The groups of processes in one of the rats are demonstrated in different colors in Figure 4. To verify that some of the observed axon growth is true dCST regeneration, we injected retrograde AAV9 in areas caudal to the C4 lesion and observed labeling of both cortical axons in the subcortical white matter and a population of corticospinal motor neurons.

We detected many mature synapses that were formed between regenerated axons below the C4 SCI and spinal neurons, including large motor neurons in the VHs. We therefore used electrophysiology to find out whether the newly regrown connected axons were functionally competent. Stimulation of the right pyramid elicited robust responses in the dorsal spinal cord above and below the lesion. These responses displayed a normal pattern in which the higher the stimulation current amplitude, the higher was the elicited electrical potential. After PI3Kδ treatment, we measured increased potential in the distal front limb flexor muscles, suggesting increased innervation, while control rats showed minimal response to stimulation. We then studied expression of the neuronal activity marker cFOS in the spinal neurons. Unexpectedly, we detected low levels of neuronal cFOS in uninjured animals similar to GFP controls in spinal neurons caudal to the lesion site. We assume that this is likely due to the prolonged time we allowed cFOS expression to be detected in treated animals. The 2.5 h we waited was probably too long for uninjured animals since cFOS peaks as early as 30 min to 1 h after stimulation in normal animals and returns to baseline after 2 h.^40^^,^^41^ Therefore, levels of cFOS were low by the time we sacrificed these rats.

### Increased plasticity, sprouting and branching, and extrapyramidal tracts

We show that overexpression of PI3Kδ in rat motor cortex resulted in functional recovery and restored connections measured by electrophysiology after SCI. It is not possible to determine definitively in our model how much of this PI3Kδ-driven recovery is due to long-distance regeneration, sprouting above the lesion, and sprouting from the intact vCST, all of which were observed as part of the PI3Kδ effect. The growth we observed was accompanied by notable branching of studied axons, which added to the complexity of the treatment effect but is not unusual in studies that achieve extensive regeneration. It has been shown that branching is a function of PI3K/PIP3 signaling in concert with mitochondria present at branch initiation sites^42^^,^^43^ and is regulated by local and temporary PTEN inhibition.^44^

The large number of regenerated axons growing in the ipsilateral and contralateral cord and their ability to form synapses suggests that they participated significantly, but sprouting above the lesion was extensive and might explain some of the recovery in the ipsilateral limbs. We believe that much of the function recovery is due to dCST regeneration because this pathway is involved in skilled reaching activity, as tested in our experiments. A contribution may also come from the extrapyramidal tracts—rubrospinal and reticulospinal^45^—although these pathways would not have been directly affected by our PI3Kδ treatment, which was only delivered to cortical neurons.^46^ The reticulospinal tract (RtST) is involved in gross front limb motor function control, arousal, muscle tone, and posture. It has been shown that RtST contributes to control of gross gripping of the upper limbs.^47^^,^^48^ We included the grip test in our behavioral assessments and observed improvements in treated rats. Additionally, we recorded EMG potentials and muscle function improvement in ipsilateral paws despite treating only one hemisphere. This is compatible with the above-lesion sprouting and the bilateral regeneration of CST axons that we observed, but there could also be a contribution via the RtST, which descends bilaterally and displays spontaneous plasticity.^49^^,^^50^ Additionally, we observed that PI3Kδ-treated rats had better posture, weight support, and muscle tone in the open field when compared to untreated controls, which would be compatible with a role for the RtST (video available in the Zenodo repository).

### Perspectives

To further increase the speed and precision target finding of axon regenerates, PI3Kδ treatment could be combined with other stimulators of regeneration and with rehabilitation. This could be achieved in combination with chondroitinase ABC, which modulates the extracellular matrix around the lesion to make it more growth permissive. Additionally, a successful regeneration promoter for ascending sensory axons from DRG into the spinal cord, able to achieve regeneration of over 4 cm, is integrin α9 combined with its activator kindlin-1. In sensory axons, which transport integrins, the combination of this tenascin-C-binding integrin and the kindlin-1 activator led to extensive regeneration for several centimeters.^51^ Because PI3Kδ restores integrin anterograde transport in cortical neurons, combining it with integrin could be more successful.^8^^,^^32^ PI3Kδ expression led to upregulation of S6 phosphorylation, which mediates feedback inhibition of the AkT/mTOR pathway. There is, however, evidence that an inhibitor of S6 phosphorylation, via S6K1, is a promoter of CST regeneration.^52^ A combination of PI3Kδ with an inhibitor of S6 phosphorylation is therefore another potentially successful combination.

## Materials and methods

### Animals

All experiments were performed in accordance with the European Communities council directive of September 22, 2010 (2010/63/EU), following the ARRIVE guidelines (https://arriveguidelines.org/), and were approved by the Ethics Committee of the Institute of Experimental Medicine ASCR, Prague, Czech Republic.

Young adult male and female Wistar or Lister Hooded rats, aged 10–12 weeks, were used in this study. Female Lister Hooded rats were used in the behavioral part of the study due to their excellent ability to learn and perform various tests, which was not possible to achieve with Wistar rats, specifically the skilled paw-reaching test. Further, Lister Hooded rats were used in tissue assessments by MRI and immunohistochemistry (IHC). Male Wistar rats were used in electrophysiology as well as tissue assessments by IHC. Rats were housed in pairs with a 12-h light/dark cycle and provided with water and food *ad libitum*. They were quarantined for 2 weeks prior to surgery, after which they were checked every day with increased care taken for the first 3–5 days, with food pellets placed inside their cage.

### Viral vector preparation

Plasmid DNA encoding AAV-CAG-eGFP and AAV-CAG-PIK3CD has been described previously,^8^ and AAV-SYN-PIK3CD (Addgene plasmid #203730) was made by VectorBuilder. Recombinant AAV vectors were produced as previously described.^53^ Plasmid DNA was scaled in-house using DH5α transformation competent cells (Invitrogen, Waltham, MA, USA) and isolated with Maxiprep (Thermo Fisher, Waltham, MA, USA). Subsequently, the presence of two ITR sites was verified using SmaI restriction enzyme digestion and agarose gel electrophoresis. Plasmids were then used to generate: AAV1-CAG-PIK3CD, titer 5 × 10^12^ gc/mL; AAV1-CAG-eGFP, titer 2.64 × 10^12^ gc/mL; and AAV1-SYN-PIK3CD, titer 2.7 × 10^12^ gc/mL. AAV1-SYN-eGFP was purchased from Vigene Biosciences with a titer of 2.3 × 10^13^ (now part of Charles River, cat. #CV17001-AV1). To avoid interference with PIK3δ activity by direct fluorescent tagging, mixtures of AAV1-sCAG-eGFP and AAV1-sCAG-PIK3CD or AAV1-SYN-eGFP and AAV1-SYN-PIK3CD were used. Each mixture contained 90% AAV1-PIK3CD and 10% AAV1-GFP. Mixtures containing AAV1-SYN-PIK3CD were compared to titer-matched AAV1-SYN-eGFP. The titer of control AAV1-GFP was therefore 2.64 × 10^11^ for AAV1-CAG-eGFP and 2.3 × 10^11^ for AAV1-hSYN-eGFP. To retrogradely label corticospinal projecting layer V neurons in intact rats, AAV2rg-hSYN-mCherry with a titer of 2 × 10^13^ gc/mL (Addgene, #114472AAV2rg) or AAV9rg-hSYN-mScarlet (AAV9rg plasmid was kindly provided by the Zhigang He lab) with a titer 5.1 × 10^13^ gc/mL was used.

### Spinal cord injury and virus injections

Rats weighing 220–400 g were anesthetized with 3% isoflurane, shaved, and placed on a heating pad that was kept at 37°C, their temperature monitored with a rectal thermometer. At this stage, buprenorphine (Bupaq Multidose 0.3 mg/mL; Richter Pharma, Austria, 0.01 mg/kg, subcutaneously [s.c.]) and caprofen (Rymadil, Pfizer; 7.5 mg/kg, intramuscularly [i.m.]) were administered as pain relief. An incision was made in the neck area and through cervical muscles to gradually expose the spine, with blunt muscle retraction used whenever possible. Laminectomy of the C4 vertebra was performed, and small punctures into the dura mater were made with a needle. A set of micro forceps (0.25 mm tip, Fine Science Tools, Foster City, CA, USA, #11083-07) with markers 1 mm from the tips was inserted into the punctures and carefully but firmly squeezed for 7 s. The 1-mm guide marks enable a lesion depth of 1 mm to the CC to be accurately judged, as shown in multiple previous publications. Animals were then sutured in anatomical layers, and skin was treated with Novikov solution.

Within the same surgery, four injections with either AAV1-hSYN-eGFP, AAV1-hSYN-eGFP + AAV1-hSYN-PIK3CD, or AAV1-CAG-eGFP + AAV1-CAG-PIK3CD were administered. Animals were fixed onto a semi-autonomous stereotaxic frame (Neurostar, Tubingen, Germany). The skull was exposed, and positions of drill and syringe were synchronized and calibrated relative to bregma and lambda. Corrections for tilt and scaling were made before drilling. Each site was then injected with 0.5-μL mixture at a rate of 0.2 μL/min at 1.5-mm depth using a 10-μL Hamilton syringe. Coordinates used were as follows: ML/AP: 1′5, 1; 2, 3′5; 3′5, 2; 3, 0′5. The needle was left in place for 3 min after injection and retracted in 0.5-mm increments every 30 s. Skin on the scalp was then sutured and treated with Novikov solution. Each rat received 2 mL of glucose solution (Ardeanutrisol, glucose 100 g/L; Ardeapharma, Sevetin, Czech Republic) at the end of surgery.

Intraspinal injections of a total of 1 μL of AAV2rg-hSYN-mCherry or AAV9rg-hSYN-mScarlet were administered to intact Wistar rats (*n* = 2 and *n* = 3, respectively) to visualize cortical layer V neurons. Rats weighing 220–400 g were anesthetized with 3% isoflurane, shaved, and placed on a heating pad that was kept at 37°C, their temperature monitored with a rectal thermometer. At this stage, buprenorphine (Bupaq Multidose 0.3 mg/mL; Richter Pharma, Austria, 0.01 mg/kg, s.c.) and caprofen (Rymadil, Pfizer; 7.5 mg/kg, i.m.) were administered as pain relief. An incision was made in the neck area and through cervical muscles to gradually expose the spine, with blunt muscle retraction used whenever possible. Laminectomy of the C4 vertebra was performed, and small punctures into the dura mater were made with a needle. A Neurostar device was used to inject 0.5 μL at one site (0.5–1 mm laterally to midline on the left side) at two depths. The needle was lowered to a depth of 0.8 mm and 0.6 mm and virus injected at a rate of 0.04 nL/min. The needle was left in place for 3 min after each injection. Upon completion and after needle removal, muscles and skin were sutured in anatomical layers, and Novikov solution was applied.

### *In vitro* MRI of the spinal cord sections and lesion size measurement

Spinal cord sections were measured when immersed in PBS solution in 2-mL test tubes. MR images were obtained using a 7-T MR scanner MRS∗DRYMAG 7.0T (MR Solutions, Guildford, UK) equipped with a mouse head resonator coil. Three sequences with high resolution were acquired. A T2-weighted turbo-spin echo sequence was initiated in axial direction, repetition time T = 4,000 ms, turbo factor TF = 8, echo spacing TE = 8 ms, effective TE = 40 ms. The number of acquisitions was 16 with acquisition time approximately 17 min. Acquired matrix was 128×, field of view (FOV) = 10 × 10 mm^2^, with 20 slices of slice thickness 0.5 mm with no gap. T1-weighted axial images were obtained using a 3D gradient echo sequence with TR = 10 ms, flip angle 20°, TE = 3.3 ms, number of acquisitions 16, and acquisition time 11 min. Matrix was 128 × 128 × 32 and FOV = 10 × 10 × 16 mm^3^ (which provides axial slices with thickness of 0.5 mm).

Lesion volumes were determined from MR images in which a lesion area was measured with ImageJ. Sums of areas were multiplied by the lesion length (number of images containing a cavity × 0.5 mm).

### Behavioral testing

Lister Hooded rats treated with AAV1-hSYN-GFP + AAV1-hSYN-PIK3CD (*n* = 15) or AAV1-hSYN-GFP (*n* = 14) only were tested with a range of behavioral tests to assess changes in functional motor and/or sensory function with a focus on front limbs. Rats were trained daily for 2 weeks before surgery to be able to perform skilled paw reaching, grip test, and ladder-rung walking. After this period, baseline measurements were recorded for all tests. After surgery and vector injections, rats were allowed a 1-week recovery break and then tested once a week for 16 following weeks.

#### Skilled paw reaching

To determine improvements in fine motor control, animals were placed into a Montoya staircase and allowed to retrieve sugar pellets for 15 min. At the start of the test, each stairwell contained three sugar pellets. After 15 min, the remaining pellets were counted, and the longest distance from which a pellet was retrieved was recorded.

#### Grip test

The grip test was used to evaluate muscular strength of rats. They were encouraged to hold on to a grid attached to a force measuring device (BIO GS3; Bioseb) with both forepaws. They were then pulled back in a horizontal plane until they lost their grip. The peak force applied to the grid was recorded just before the loss of grip. This was repeated five times, and values were noted. Only the three highest values were averaged and used for analysis.

#### Ladder-rung walking

A ladder with unevenly spaced rungs was used, and rats were recorded traversing the ladder five times. Animals were encouraged to cross with sugar pellets, and their stepping was evaluated according to Metz and Whishaw.^54^ Each score was then normalized to the number of steps evaluated per crossover.

#### Von Frey sensory test

The Von Frey test was used to determine differences in sensitivity to mechanical noxious stimuli. The device used consisted of transparent compartments where rats were placed standing on a mesh platform through which von Frey fibers were applied to the plantar surface of each forepaw (IITC Life Science, CA, USA). Prior to the test, rats were placed into the compartments and allowed to acclimatize for 15 min. Each forepaw was measured five times, and the value upon paw withdrawal was recorded. Care was taken to ensure the rat was unaware of the fiber being applied. For statistical analysis, the upper and lower extreme values were omitted before averaging the remaining three values.

### Electrophysiology recording

In a terminal setting, Wistar rats treated with PI3Kδ (*n* = 12) or GFP vectors (*n* = 9) surviving for 16 weeks after injury and treatment, and uninjured controls (*n* = 6), were anesthetized with urethane (1.5 g/kg). An incision was made in the neck and back areas to gradually work through the muscle layers and expose the obex by removing the cerebellar tonsil as described previously.^55^^,^^56^ Subsequently, the cervical spinal cord including the lesion and up to 1 cm below was also exposed. A stimulating tungsten electrode with impedance 100 kΩ was lowered into the right pyramid. Concurrently, a stationary silver ball electrode was placed on the cord surface 1 cm cranially to the lesion site as a control of correct stimulating electrode placement. Another silver ball electrode was placed 1 cm below the lesion 1 mm laterally from midline to the left. Silver ball electrodes were used to measure cord dorsum potentials (CDPs). Within the same setting, subcutaneous silver electrodes were inserted into the distal forearm muscles of both left and right forearms for EMG recordings. Potentials were evoked via stimulation with five square-wave pulses at 300 Hz, 30–300 μA and 400 μs, delivered every 0.33 s as used previously.^57^ Responses were recorded 3–5 times, with each response approximating 50 stimulations. Once all measurements were completed, a re-lesion was performed to confirm loss of response and to validate recordings. Rats were then maintained for 2.5 h to allow for cFOS upregulation.

### Immunohistochemistry

Floating 40-μm frozen brain coronal sections, 20-μm mounted spinal cord cross-sections, or 20-μm sagittal/frontal sections were washed with Tris-buffered saline (TBS). When staining for PI3Kδ, a heat-induced antigen retrieval (HIER) step was incorporated into the protocol. Sections were washed once in dH_2_O and transferred into a 4.5 mM aqueous solution of citraconic anhydride. Plates containing the samples were placed in a water bath and warmed to 98°C for 20 min. Samples were then left to cool completely before washing once in dH_2_O and twice in TBS before continuing with standard protocols. Work with the HIER agent was performed under a fume hood. Sections were then permeabilized with Triton X-100 for 20 min and endogenous avidin/biotin blocked with avidin/biotin block (Abcam, Bristol, UK; #ab64212) in samples where biotin-streptavidin amplification was used before blocking with 10% chemiBLOCKER (Merck Millipore, Billerica, MA, USA; #2170) for 2 h at room temperature. Following incubation in primary antibody, samples were washed and incubated in their respective secondary antibody (1:400, Life Technologies, Carlsbad, CA, USA) or biotinylated secondary antibody (1:400, Vector Biotechnologies) for 2 h at 4°C, then washed and incubated in Streptavidin Alexa Fluor 488/594 (1:400, Life Technologies) at 4°C for an additional 2 h and DAPI (1:3,000) for 10 min. After washing, sections were mounted with Vectashield Antifade Mounting Medium (Vector Laboratories, CA, USA; H-1000-10). Primary antibodies used in this study were rabbit anti-p110 (1:300, Abcam, #ab1678), chicken anti-GFP IgY fraction (1:400, Thermo Fisher Scientific, #A10262), mouse anti-pS6 (1:300, Cell Signaling, Danvers, MA, USA, #62016), mouse anti-PKCγ (1:1,000, Santa Cruz, Dallas, TX, USA, sc166451 A-7 clone), mouse anti-cFOS (1:500, Abcam, #ab208942), rabbit vGlut1/2 (1:500, Synaptic Systems, Göttingen, Germany, #135503), guinea pig Homer1 (Synaptic Systems, #160004), and mouse NeuN (Merck, EMD Millipore, Darmstadt, Germany, #MAB377). Images of cross-sections 1 cm below lesion to visualize vGlut1/2 together with GFP were acquired with an Andor Dragonfly 503 spinning-disk confocal microscope (Oxford Instruments, Abingdon, UK) (Figure S3).

#### Co-expression analysis, pS6, and soma size

Co-expression of PI3Kδ and GFP was determined in 4–5 brain coronal sections per rat 12 weeks (*n* = 4) and 16 weeks (*n* = 8) after surgery using Imaris 9.4 software. Images were obtained using a confocal microscope at 20× magnification (Olympus, SpinSR10). First, PI3Kδ^+^ cells were identified from which a PI3Kδ^+^GFP^+^ fraction was determined. Levels of phosphorylated S6 protein were determined in 3–6 brain coronal sections per rat in the same fashion as co-expression analysis in which layer V PI3Kδ^+^ neurons were identified and a PI3Kδ^+^pS6^+^ fraction determined.

Using ImageJ (NIH, Bethesda, MD, USA), neural soma length of layer V cortical neurons was determined in five sections per rat in immunostained neurons expressing PI3Kδ, as annotated with white lines in Figure 1, after 12 weeks (*n* = 4) and 16 weeks (*n* = 4) and compared with lengths from respective control groups expressing GFP only (*n* = 3 and *n* = 3) and from intact animals expressing retrogradely delivered mCherry (*n* = 2). One-way ANOVA with Tukey’s post hoc tests were used.

#### PI3Kδ expression level analysis

Comparison of mean fluorescent intensity (MFI) of PI3Kδ between AAV1-CAG-PI3Kδ and AAV1-hSYN-PI3Kδ was determined in three brain coronal sections per rat (*n* = 3). Images were obtained using a confocal microscope at 20× magnification (Olympus, SpinSR10). Using ImageJ, the PI3Kδ-positive region was selected and MFI analyzed, and values were normalized to area. Unpaired t test was used in statistical analysis.

#### Axon sprouting analysis

To quantify the number of axons sprouting above the lesion following C4 dorsal column crush, we used spinning-disk microscopy (20× magnification) to image cervical spinal cord cross-sections (5–6 sections per animal) at 100-μm increments. The number of GFP^+^ axons were counted using ImageJ, by an observer blind to experimental groups. In brief, a horizontal line was drawn from the CC to the lateral gray and white matter border, then an average of ten vertical lines spaced 100 μm apart were used to count axons in the contralateral gray matter of each 100-μm section. Differences in the number of axons in VHs and DHs were also observed, and all values were normalized to the MFI of the dCST (average from three images per rat) and area of DHs/VHs. We also analyzed cross-sections from rats injected with vectors carrying transgenes under the CAG promoter. The number of GFP^+^ axons was averaged across the 5–6 images and compared between experimental groups (*n* = 3–4 per group) using GraphPad prism software, presented as mean ± SEM. One-way ANOVA with Tukey’s post hoc tests were used.

#### Retraction bulbs analysis

Sagittal sections stained against GFP were used to determine the number of RBs and their distance from the lesion border. We used a confocal microscope at 40× magnification (Olympus, SpinSR10) to image three sections per rat that clearly contained the dCST, after which ImageJ was used to count RBs and their distance from lesion to compare the level of axon degeneration in AAV1-hSYN-PIK3CD- and AAV1-hSYN-eGFP-treated rats (*n* = 3 and *n* = 3, respectively) at 16 weeks. A t test with Mann-Whitney post hoc test was used.

#### Axon counting and regeneration index

Every other sagittal 20-μm section was used for staining against GFP and axon counting in PI3Kδ-treated rats and their controls 12 weeks (*n* = 4 and 3) and 16 weeks (*n* = 4 and 5) after SCI and cortical injections. A grid with 60-μm spacing placed in a microscope eyepiece allowed us to determine the distance below the lesion. A count of axons every 60 μm was determined using an Axioskop 2 plus microscope (Zeiss, Oberkochen, Germany). At each 60-μm increment, the number of axons crossing a line was noted. Axons appearing to be part of the vCST, which are not destroyed in this model, were not counted. Axons clearly sprouting from the vCST were also not included. For plotting reasons, sums of axons of every 600-μm increment were used, which were a product of a sum of all sections counted that corresponded to the same caudal distance from the lesion. A regeneration index was then calculated as the number of counted axons normalized to the mean number of axons in the dCST counted in three cross-sections above the lesion. A paired t test was used to calculate significant differences between groups. A reconstruction of regenerated spinal cord was produced by approximate drawing of GFP^+^ axons and neurites. Blue color was assigned to those found cranially to lesion and extending, red color was assigned to axons and neurites in the ventral part of sections, and green color represented neurites residing in the dorsal half of the cord caudal to the lesion border (Figure 4C).

#### Qualitative analysis of synapses below the lesion site

Spinal cross-sections from animals 16 weeks after treatment and stained for neuronal marker NeuN (Alexa 647), regenerated axon marker GFP, presynaptic vGlut1/2, and postsynaptic Homer1 were imaged at 40× and 63× oil-immersion magnification using an Olympus SpinSR10 confocal microscope. Individual planes of each acquired z stack were examined for evidence of mature synapses formed between regenerated axons and spinal neurons. Representative images of indicated planes were selected for Figure 5 from areas near the CC (Figure 5A) and VH (three different planes, Figure 5B).

#### cFOS analysis

Spinal cross-sections from rats examined in the electrophysiology part of this study (PI3Kδ treated *n* = 11, GFP controls *n* = 5, uninjured *n* = 5) from 0.5 cm above lesion site and 1 cm below lesion were stained for cFOS, an early marker of neuronal activity.^58^ This analysis was optimized in regenerating rats. Three rats were excluded from analysis because they had to be euthanized before they were suitable for cFOS analysis, due to the required survival time after stimulation. Images of three sections per rat were taken with an epifluorescence microscope (Leica, CTR6500) equipped with TissueFAXS 4.2.6245.1020 software (TissueGnostics, Vienna, Austria) and were then analyzed with ImageJ in five regions: central canal area (CC), contralateral ventral horn (VH1 contra), contralateral dorsal horn (DH1 contra), ipsilateral ventral horn (VH2 ipsi), and ipsilateral dorsal horn (DH2 ipsi). The number of cFOS^+^ nuclei was then normalized to the selected area size. One-way ANOVA or unpaired t test was used in statistical analysis.

## Data and code availability

Data are available at the Zenodo repository: https://doi.org/10.5281/zenodo.11518402.

## Acknowledgments

We would like to thank Prof. Britta Eickholt for her critical reading of the manuscript and her useful feedback. This research was supported by the 10.13039/501100001708International Foundation for Research in Paraplegia (IRP) P186, OPJAK EXREGMED CZ.02.01.01/00/22_008/0004562, 10.13039/501100000265Medical Research Council (MR/V002694/1), 10.13039/501100000265UK Medical Research Council (G105497), Fight for Sight grant 5065-5066, and KNAW research fund; and by the Microscopy Service Centre of the Institute of Experimental Medicine CAS supported by the MEYS CR (LM2023050 Czech-Bioimaging), which includes support by the Ministry of Education, Youth and Sports of the Czech Republic (Research Infrastructure NanoEnviCZ, LM2018124) and the 10.13039/501100000780European Union – European Structural and Investment Funds in the frame of the Research Development and Education project Pro-NanoEnviCZ operational program (project no. CZ.02.1.01/0.0/0.0/16_013/0001821). Further, MRI imaging work was supported by the European Regional Development Fund project “Modernization and support of research activities of the national infrastructure for biological and medical imaging Czech-BioImaging” (no. CZ.02.1.01/0.0/0.0/18_046/0016045). For Open Access, a CC BY 4.0 public copyright license is applied to any Author Accepted Manuscript (AAM) version arising from this submission.

## Author contributions

K.K. designed and conducted the experiments, produced viral vectors, analyzed data, and wrote the manuscript. Z.P. conducted experiments and analyzed data. L.K. acquired and analyzed data. S.S. conducted experiments and analyzed data. B.N. designed and produced viral vectors and edited the manuscript. R.H. conducted and analyzed experiments. V.H. acquired and analyzed data from MR imaging and secured funding. L.M.U. participated in supervision, funding acquisition, and experimental design. R.T. designed experiments and provided electrophysiology tools. J.C.K. supervised and designed experiments and secured funding. J.v.d.H. produced viral vectors. J.V. designed viral vector production, provided supervision, and reviewed the manuscript. R.E. designed experiments, secured funding, and edited the manuscript. J.W.F. conceptualized the project, designed experiments, secured funding, provided mentorship, and wrote the manuscript. P.J. conceptualized the project, designed experiments, secured funding, provided mentorship, and edited the manuscript.

## Declaration of interests

The authors declare no competing interests.

## Supplemental information

Document S1. Figures S1–S4

Document S2. Article plus supplemental information
