# Supplementary material for: Hyperactive delta isoform of PI3 kinase enables long-distance regeneration of adult rat corticospinal tract
Source: Mol Ther. 2025 Jan 1;33(2):752–70. doi: 10.1016/j.ymthe.2024.12.040 (PMC11852985; doi:10.1016/j.ymthe.2024.12.040)
Supplement: Document S1. Figures S1–S4 [file mmc1.pdf]

YMTHE, Volume *33*

Supplemental Information

Hyperactive delta isoform of PI3 kinase enables long-distance regeneration of adult rat corticospinal tract

Kristyna Karova, Zuzana Polcanova, Lydia Knight, Stepanka Suchankova, Bart Nieuwenhuis, Radovan Holota, Vit Herynek, Lucia Machova Urdzikova, Rostislav Turecek, Jessica C. Kwok, Joelle van den Herik, Joost Verhaagen, Richard Eva, James W. Fawcett, and Pavla Jendelova

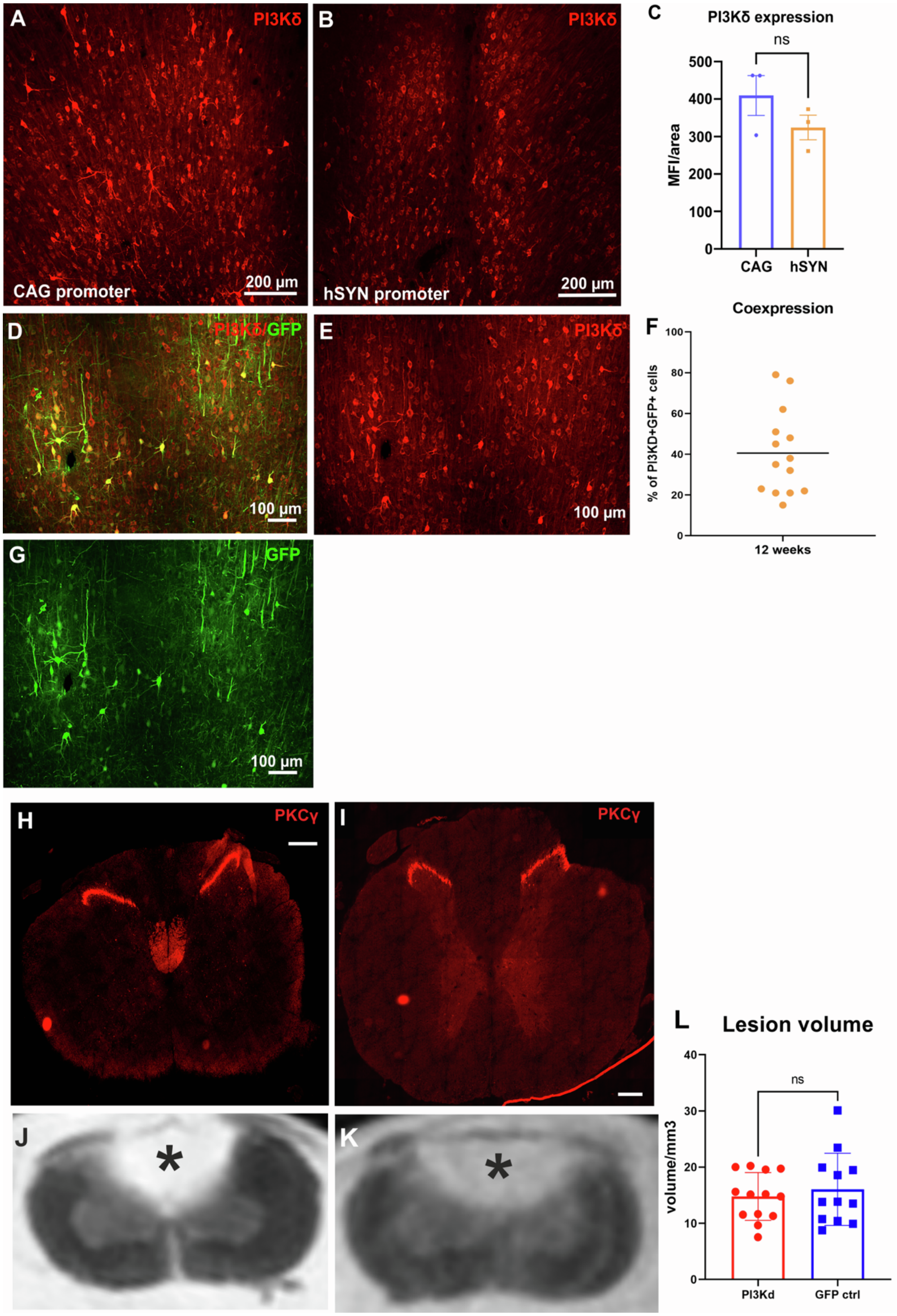

Figure S1. Microscopy images and expression analysis, MRI and lesion volume, related to Figures 1 and 4. A comparison of PI3Kδ expression levels in cortices of rats treated with AAV1-CAG-PIK3CD (A) and AAV1-hSYN-PIK3CD (B). The mean fluorescence intensity (MFI) was higher after treatment with AAV1-CAG-PIK3CD but did not reach statistical significance (C). Coexpression of PI3Kδ and GFP after treatment with AAV1-CAG vectors was also assessed and determined to be approximately 40% (D-G). Lesion completeness was confirmed by methods of either PKCγ staining (Wistar) of the dorsal CST above (H) and below (I) lesion, or by an MRI scan of the whole cord (Lister Hooded) in both PI3Kδ treated (J) or control rats (K). Lesion volumes determined from MRI imaging were of similar volumes in both treated and control groups (L). Lesion annotated with asterisk. Scale bars 100 µm. Unpaired t-test was used.

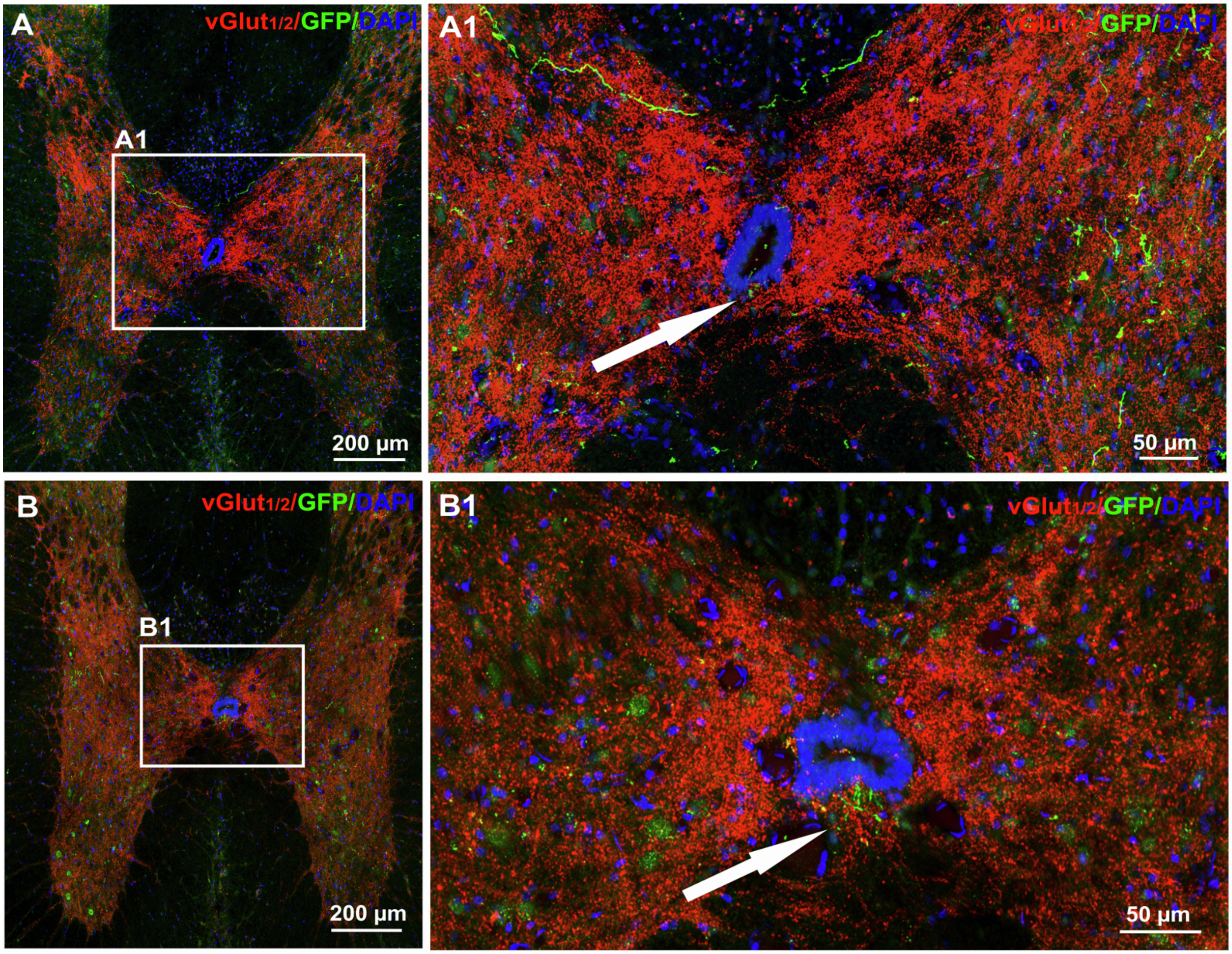

Figure S2. Microscopy images, related to Figures 4 and 5. Cross sections from 2 different rats (A, B) showing the central canal area (A1, B1) both with axons in the gray matter right below the central canal approximately 1 cm caudally to the lesion border. These axons are annotated with white arrows.

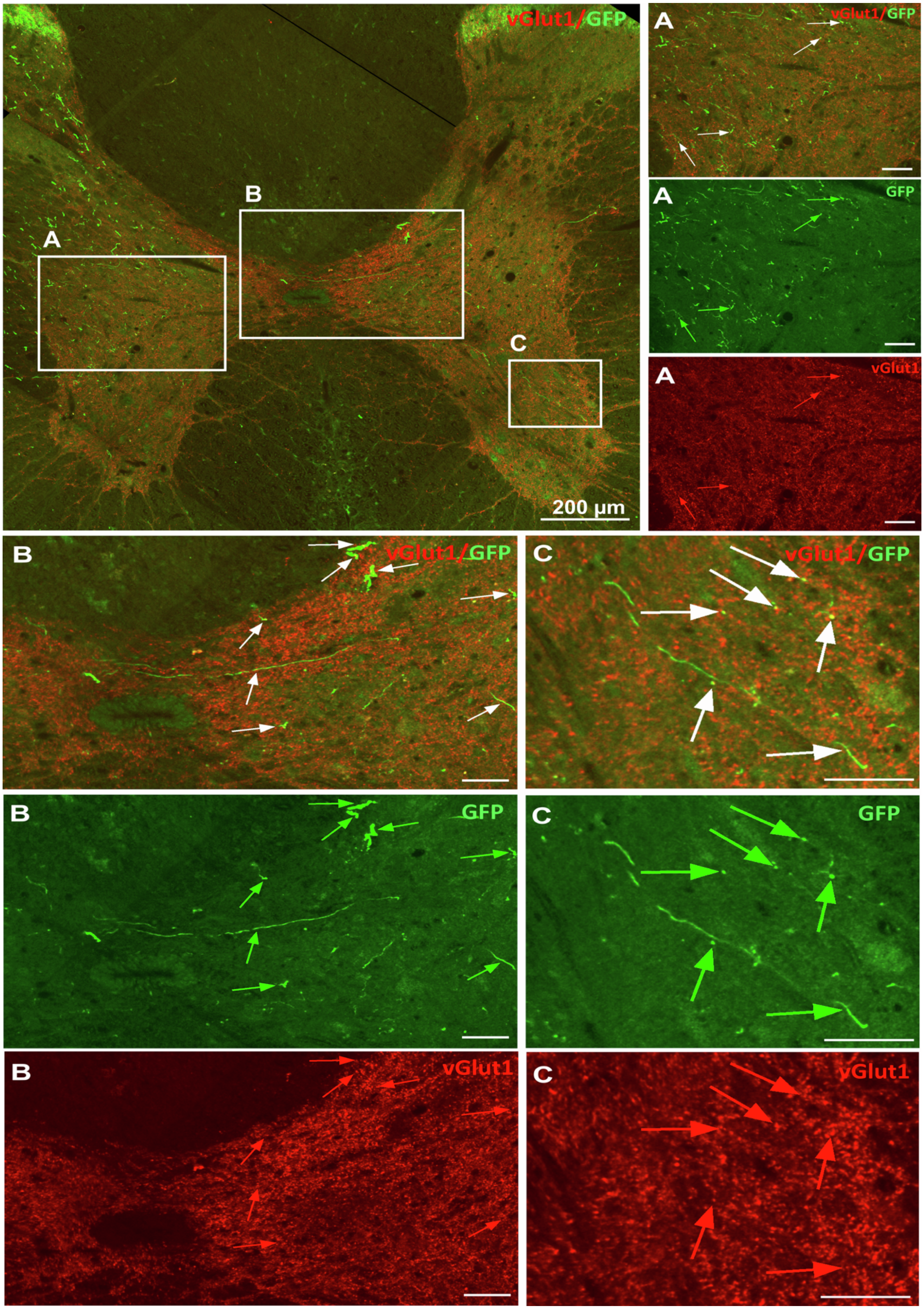

Figure S3. Microscopy images, related to Figure 5. In 20 µm cross sections, GFP+ axons below lesion colocalize with a vGlut1/2 excitatory synapse markers. Examples shown in ventral horn areas (A, C) and near the central canal (B). Images were acquired with Dragonfly spinning disc microscope at 40x magnification. Scale bars 50 µm unless otherwise stated.

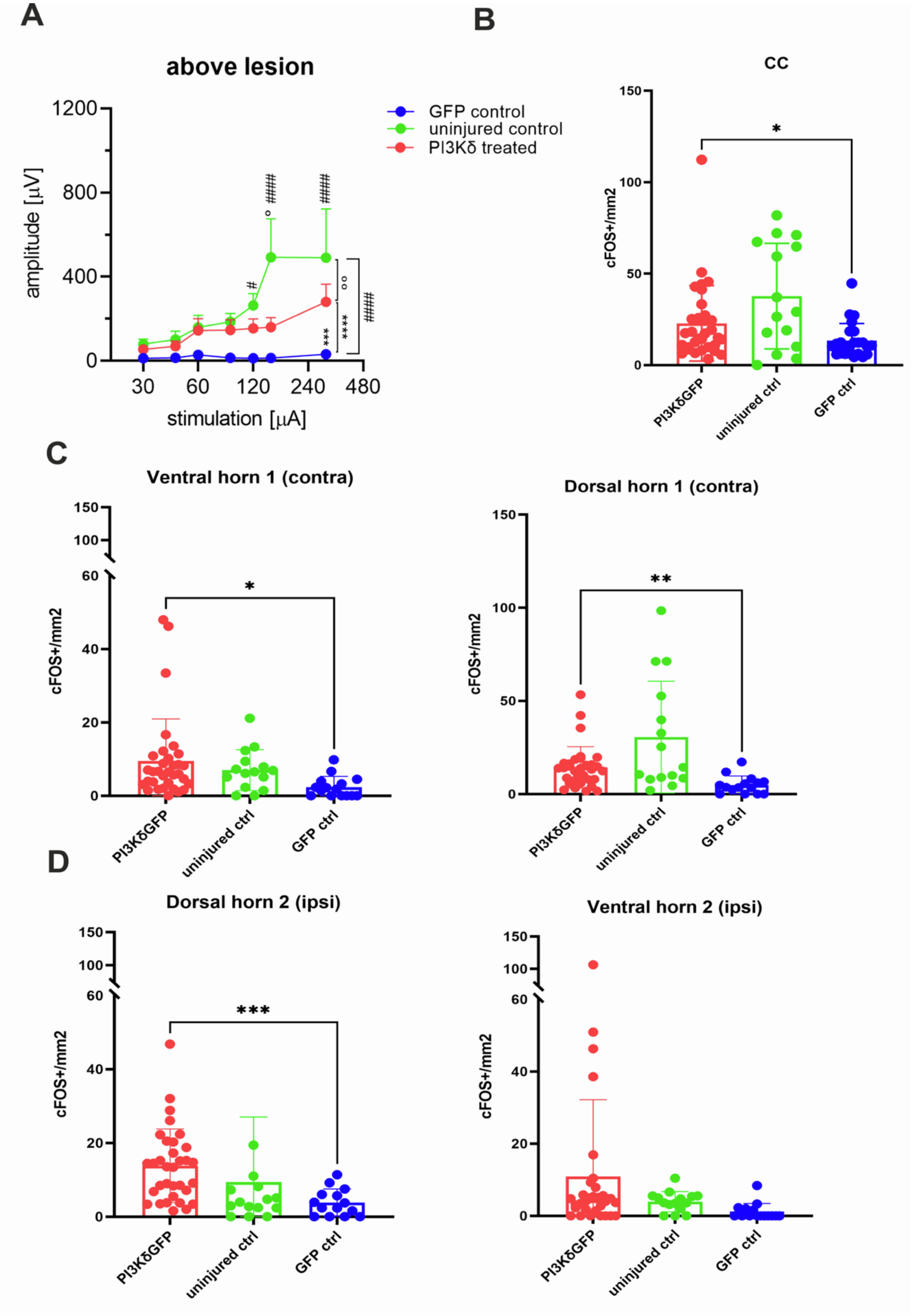

Figure S4. Electrophysiology and cFOS analysis cranially to the lesion, related to Figures 7 and 8. CDP measurements from above the lesion were recorded and used as guidance of proper electrode placement. All measured rats elicited responses from this area, but they were significantly smaller in controls when compared to other groups (A). $cFOS^{+}$ nuclei densities in spinal cord gray matter shows higher values or neural activation in AAV1-hSYN-PIK3CD treated rats around central canal (B), contralateral ventral and dorsal horns (C). and in ipsilateral dorsal horn (D) when compared to AAV1-hSYN-eGFP controls.
